# Opportunistic Screening With Low-Dose Computed Tomography and Lung Cancer Mortality in China

**DOI:** 10.1001/jamanetworkopen.2023.47176

**Published:** 2023-12-12

**Authors:** Lijie Wang, Yue Qi, Ailing Liu, Xiaolei Guo, Shanshan Sun, Lanfang Zhang, Huaijun Ji, Guiyuan Liu, Huan Zhao, Yinan Jiang, Jingyi Li, Chengcun Song, Xin Yu, Liu Yang, Jinchao Yu, Hu Feng, Fujun Yang, Fuzhong Xue

**Affiliations:** 1 Department of Biostatistics, School of Public Health, Cheeloo College of Medicine, Shandong University, Jinan, China; 2 Healthcare Big Data Research Institute, School of Public Health, Cheeloo College of Medicine, Shandong University, Jinan, China; 3 Department of Endocrinology, Shandong Provincial Hospital Affiliated to Shandong First Medical University, Jinan, China; 4 Department of Oncology, Weihai Municipal Hospital, Cheeloo College of Medicine, Shandong University, Weihai, China; 5 Department of Pulmonary and Critical Care Medicine, Weihai Municipal Hospital, Cheeloo College of Medicine, Shandong University, Weihai, China; 6 Department for Chronic and Non-Communicable Disease Control and Prevention, Shandong Center for Disease Control and Prevention, Jinan, China; 7 Department of Chemotherapy, Weihai Municipal Hospital, Cheeloo College of Medicine, Shandong University, Weihai, China; 8 Department of Thoracic Surgery, Weihai Municipal Hospital, Cheeloo College of Medicine, Shandong University, Weihai, China; 9 Department of Radiology, Weihai Municipal Hospital, Cheeloo College of Medicine, Shandong University, Weihai, China; 10 Department of Radiotherapy, Weihai Municipal Hospital, Cheeloo College of Medicine, Shandong University, Weihai, China; 11 Qilu Hospital, Cheeloo College of Medicine, Shandong University, Jinan, China

## Abstract

**Importance:**

Despite the recommendations of lung cancer screening guidelines and the evidence supporting the effectiveness of population-based lung screening, a common barrier to effective lung cancer screening is that the participation rates of low-dose computed tomography (LDCT) screening among individuals with the highest risk are not large. There are limited data from clinical practice regarding whether opportunistic LDCT screening is associated with reduced lung-cancer mortality.

**Objective:**

To evaluate whether opportunistic LDCT screening is associated with improved prognosis among adults with lung cancer in mainland China.

**Design, Setting, and Participants:**

This cohort study included patients diagnosed with lung cancer at Weihai Municipal Hospital Healthcare Group, Weihai City, China, from 2016 to 2021. Data were analyzed from January 2022 to February 2023.

**Exposures:**

Data collected included demographic indicators, tumor characteristics, comorbidities, blood indexes, and treatment information. Patients were classified into screened and nonscreened groups on the basis of whether or not their lung cancer diagnosis occurred through opportunistic screening.

**Main Outcomes and Measures:**

Follow-up outcome indicators included lung cancer–specific mortality and all-cause mortality. Propensity score matching (PSM) was adopted to account for potential imbalanced factors between groups. The associations between LDCT screening and outcomes were analyzed using Cox regression models based on the matched data with correction for lead time and length biases. Propensity score regression adjustment and inverse probability treatment weighting were used for sensitivity analysis.

**Results:**

A total of 5234 patients (mean [SD] baseline age, 61.8 [9.8] years; 2518 [48.1%] female) with complete opportunistic screening information were included in the analytical sample, with 2251 patients (42.91%) receiving their lung cancer diagnosis through opportunistic screening. After 1:1 PSM, 2788 patients (1394 in each group) were finally included. The baseline characteristics of the matched patients were balanced between groups. Opportunistic screening with LDCT was associated with a 34% lower risk of lung cancer death (HR, 0.66; 95% CI, 0.54-0.80) and 28% lower risk of all-cause death (HR, 0.72; 95% CI, 0.60-0.86) after correction for lead time and length biases.

**Conclusions and Relevance:**

In this cohort study of patients with lung cancer, opportunistic lung cancer screening with LDCT was associated with lower lung cancer mortality and all-cause mortality. These findings suggest that opportunistic screening is an important supplement to population screening to improve prognosis of adults with lung cancer.

## Key Points

**Question:**

Is opportunistic lung cancer screening with low-dose computed tomography (LDCT) associated with lower lung cancer mortality?

**Findings:**

In this cohort study of 5234 adults with lung cancer, opportunistic screening with LDCT was significantly associated with improved prognosis of adults with lung cancer in mainland China.

**Meaning:**

These findings suggest that opportunistic lung cancer screening was associated with lower lung cancer mortality and may be an important supplement to population screening.

## Teaser

This cohort study evaluates the association of opportunistic low-dose computed tomography (LDCT) screening with improved prognosis of adults with lung cancer in mainland China.

## Introduction

Lung cancer has become the second most commonly diagnosed malignant tumor and the leading cause of cancer-related death worldwide.^1^ Lung cancer may not cause any symptoms in early stages. Signs and symptoms of lung cancer typically appear only when the disease becomes severe and advanced, and treatments for late-stage lung cancer usually have a poor prognosis, with a 5-year survival rate less than 10%.^2,3,4^ Therefore, timely diagnosis of lung cancer at an early stage is an important measure to improve patient survival.

Results from the US-based National Lung Cancer Screening randomized clinical trial^5^ showed that screening for lung cancer in individuals with high risk using low-dose computed tomography (LDCT) reduced mortality from lung cancer by 20%. After that, many health organizations began calling on governments and international institutions to offer lung cancer screening through LDCT to high-risk populations (eg, heavy smokers), and corresponding lung cancer screening guidelines were issued.^6,7,8,9,10,11,12,13^ A Chinese guideline for the screening and early detection of lung cancer recommends LDCT screening for people aged 50 to 74 years who meet any of the following conditions, including heavy smoking, passive smoking, chronic obstructive pulmonary disease, occupational exposure history, and family history of lung cancer.^14^ A 2022 study by Li et al^15^ showed that 1-off LDCT screening was associated with significantly lower lung cancer mortality and all-cause mortality in a large population-based cohort in China.

Although many studies have demonstrated that policy- or program-driven population-based LDCT screening is associated with reduced lung cancer mortality, the obvious challenge is that the coverage and participation rates of individuals at high risk receiving LDCT screening are limited, resulting in lower screening efficiency.^16,17,18^ Another type of screening is clinical opportunistic screening jointly determined by physicians and patients, such as patients undergoing routine health examinations. The Healthy China Initiative 2019-2030 program is the country’s signature national domestic health policy and clearly states that for cancers with high incidence and relatively mature screening measures, such as lung cancer, local governments should promote universal opportunistic cancer screening according to the cancer epidemiology in the region.^19^

Like population screening, there are 2 indicators to evaluate the effectiveness of opportunistic screening, (1) increasing the detection rate of early cancer and (2) reducing cancer mortality and all-cause mortality. A 2013 study by Kim et al^20^ reported that the proportion of early gastric cancer detected in population-based screening was higher than that detected in opportunistic screening (74.0% vs 53.8%; *P* = .046). A 2015 study in Denmark by Mette et al^21^ found that compared with systematic population screening by cervical cytology, women undergoing opportunistic screening were more likely to have anomalous cytological findings. A study by Ke et al^22^ found that endoscopic opportunistic community-based screening was associated with a significant 66% (95% CI, 19%-86%) reduction in esophageal cancer mortality. To our knowledge, whether opportunistic screening for lung cancer is associated with reduced cancer mortality lacks systematic research evidence and therefore needs more attention.

Lung cancer has shown a trend of increasing incidence from west to east in China. Weihai City in northeastern China has a high lung cancer incidence.^23^ Weihai Municipal Hospital Healthcare Group is the largest health care group in Weihai City with the widest range of services, covering 4 districts (Rushan, Huancui, Rongcheng, and Wendeng).

Propensity score methods are widely used to control for baseline confounding by balancing baseline covariates between different treatment groups when examining the associations of treatment with outcomes in observational studies.^24^ This study was conducted to evaluate whether opportunistic screening was associated with improved prognosis among adults with lung cancer based on the Weihai regional lung cancer cohort using propensity score matching. After initial publication and in response to readers’ comments, we undertook to re-analyze the data. Given that the study participants all had lung cancer, this re-analysis included addressing lead time and length biases.

## Methods

### Study Design and Objects

This retrospective cohort study was approved by the Public Health Ethics Committee of Shandong University, and written informed consent was waived for all participants because of the retrospective nature of the study. This cohort study followed the Strengthening the Reporting of Observational Studies in Epidemiology (STROBE) reporting guideline.

Patients with newly diagnosed lung cancer at Weihai Municipal Hospital Healthcare Group, Weihai City, Shandong Province, China, from January 2016 to May 2021, were enrolled as the lung cancer cohort. The inclusion criteria were histopathologically confirmed lung cancer (*International Statistical Classification of Diseases and Related Health Problems, Tenth Revision* [*ICD-10*] code: C34) either by surgery specimen or by biopsy, including bronchoscopic biopsy, lung biopsy, or lymph node biopsy and age 18 years or older. The patients were excluded if they had a secondary lung cancer diagnosis, past medical history of malignant neoplasms, or missing or invalid identification. Patients were classified into screened and nonscreened groups on the basis of whether or not their lung cancer was diagnosed through opportunistic screening. Opportunistic screening in this study was defined as real clinical circumstances jointly determined by physicians and participants, such as a health check-up, nonpulmonary visits, and annual visits, and opportunistic LDCT was offered to all such patients.

### Ascertainment of Exposures

For each patient, 87 variables were collected from the electronic medical record: demographic characteristics, tumor characteristics, comorbidities, baseline blood indices, and treatment modalities (eTable 1 in Supplement 1). Ethnicity was determined by self-report and categorized as Han and non-Han, such as Bai and Yao. Ethnicity was included in analysis because it may be associated with lung cancer risk. The principles for selecting variables were that the missing rates of the variables were less than 20%, with 71 of 87 variables having missing values. Detailed information for missingness is shown in eFigure 1 and eFigure 2 in Supplement 1. The second reason for the variables select was that included comorbidities were limited to those that have been consistently associated with lung cancer risk from the literature and clinical experts’ experience. The TNM stage was determined according to the definition of the eighth TNM stage classification for lung cancer (eTable 2 in Supplement 1). The *ICD-10* codes corresponding to the included comorbidities are shown in eTable 3 in Supplement 1. The blood indices were categorized into quartiles, and among them, 7 blood indices (eg, red blood cell count) were sex-defined quartiles because the reference values for these indicators differ between sexes (eTable 4 in Supplement 1).

### Follow-Up

The study outcome indicators were lung cancer–specific mortality and all-cause mortality. We linked to the database of death registration and medical insurance of Shandong Province by civil identification number. Overall survival was defined as the interval between the first diagnosis of lung cancer and the date of death or December 1, 2021, whichever came first.

### Statistical Analysis

Basic characteristics of the data are presented as means with SDs for continuous variables and frequency and percentages for categorical variables. The *t* test was applied to compare the differences between groups of continuous variables, and the χ^2^ was used for categorical variables. The standardized mean difference (SMD) was also applied to the equilibrium comparison of both continuous variables and categorical variables.

The data were first imputed by multiple imputation in which the missing data were filled in 5 times. Then, propensity score matching (PSM) was applied to estimate the association of opportunistic screening (treatment) with mortality (outcomes) for lung cancer. First, using LDCT screening as the dependent variable, all pretreatment covariates measured at baseline, including demographic characteristics, tumor characteristics, comorbidities, and baseline blood indices, were included in the L1-regularized least absolute shrinkage and selection operator logistic regression to get the adjusted set of covariates and generate the probability of LDCT screening, namely, propensity score.^25,26^ According to studies reporting lead-time adjusted survival analysis,^27,28,29^ we excluded stage in the PSM model. Including this along with lead-time correction would likely obscure the effect of screening on mortality by overcorrecting benefits from stage shift. Second, we plotted the distribution of propensity scores and preference scores of the screened and nonscreened groups to ensure that there was a matching population. Third, the matched samples were obtained by performing nearest-neighbor matching, with a caliper set at 0.2 SD of the logit of the propensity score in a 1:1 ratio between groups. Fourth, balance diagnosis was carried out on the matched data, and the absolute value of SMDs of each covariate were calculated. It was considered a successful match if the absolute SMD of each covariate was controlled within 10%. Fifth, based on the matched data, the univariate Cox regression model of LDCT opportunistic screening in lung cancer mortality and all-cause mortality was fitted to obtain the hazard ratio (HR) and 95% CI of opportunistic screening for the corresponding outcomes. Propensity score regression adjustment and inverse probability treatment weighting, as well as stratified analyses by TNM stage were conducted to examine the robustness of our results.

To acknowledge the potential impact of lead time and length biases, we used methods proposed by Duffy et al.^30^ Accordingly, to correct for lead time bias, we subtracted the expected lead time from the observed survival time for each case in the screened group. Towards this, we note large variation in reported lead times in the literature.^31,32^ We therefore focused on an intermediate lead time of 210 days and comprehensively studied 150, 180, 240, and 270 days for sensitivity analysis (see eMethods in Supplement 1). To correct for length bias, we first followed Duffy et al’s method to calculate the unadjusted for confounding HR with and without correcting for length bias, which depends on the anticipated lead time bias. These results were used to compute a length bias correction factor, which was then applied to the HR estimates obtained after correction for confounding and lead time biases. By employing this combined, ad-hoc approach, all the 3 of biases were addressed in the final HR estimates.

All analyses were implemented using R statistical software version 4.1.3 (R Project for Statistical Computing). All tests were 2-sided, and *P* < .05 was considered statistically significant. The SMD greater than 0.1 was considered statistically significant. Data were analyzed from January 2022 to February 2023.

## Results

### Basic Characteristics of the Cohort

**Figure 1.**
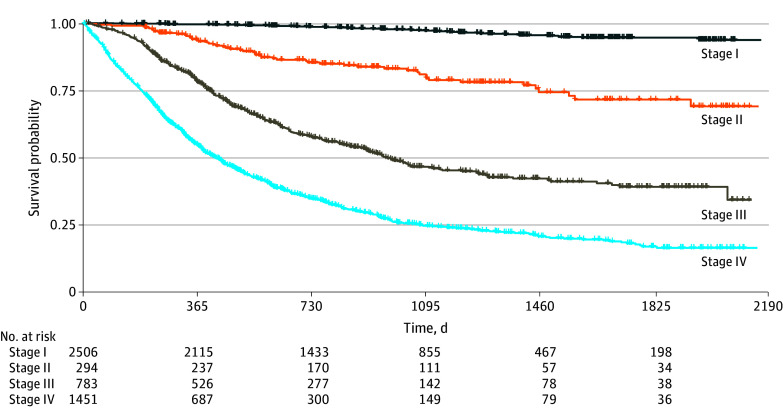
Kaplan-Meier Curve of Survival Probability of the Total Cancer Cohort by TNM Stage

The lung cancer cohort included 5246 patients (mean [SD] baseline age, 61.82 [9.84] years; 2521 [48.06%] female), with 1539 deaths attributed to lung cancer and 1666 deaths from all causes as of December 1, 2021. The median (IQR) follow-up time was 1.6 (0.9-3.0) years, and the longest follow-up time was 5.9 years. The 5-year survival rate estimated using the Kaplan-Meier method^33^ was 59% (95% CI, 57%-61%), with no median survival time observed. Most patients (4715 patients [89.85%]) had non–small cell lung cancer. There were 2506 patients (47.77%) with stage I cancer, of whom 2207 patients had stage IA, and 294 patients (5.60%) had stage II cancer, 783 patients (14.92%) had stage III cancer, 1451 patients (27.66%) had stage IV cancer, and 212 patients (4.04%) had unknown cancer stage. The Kaplan-Meier curve of the survival probability according to the TNM stage is shown in Figure 1.

### Comparison of Characteristics Between Opportunistic Screening and Nonopportunistic Groups

**Figure 2.**
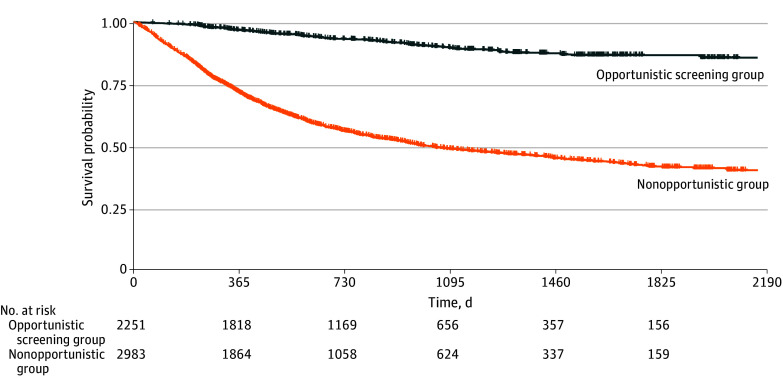
Kaplan-Meier Curve of Survival Probability According to Whether the Patients Received Their Lung Cancer Diagnosis via Opportunistic Screening

A total of 5234 patients with lung cancer (mean [SD] age, 61.81 [9.85] years; 2518 [48.11%] female) were included in the unmatched comparison between the opportunistic and nonopportunistic screening groups (excluding 12 patients with unknown screening status). Among them, 2251 patients (42.91%) received their lung cancer diagnosis through opportunistic screening and 2983 patients (56.86%) received their lung cancer diagnosis due to relevant symptoms and signs, such as chest pain, and hemoptysis. The survival rate of lung cancer in the opportunistic screening group was significantly higher than that in the nonopportunistic group (χ^2^ = 830.8; *P* < .001) (Figure 2).

As shown in Table 1 and eTable 5 in Supplement 1, a total of 79 variables were statistically significantly different between the opportunistic screening group and nonopportunistic group. Compared with patients in the nonopportunistic group, patients in opportunistic screening group were younger and were more likely to have family history of lung cancer (Table 1). The proportions of patients who were male, had medical insurance for urban and rural residents, smoked, and had respiratory diseases were significantly lower in the opportunistic screening group (Table 1).

**Table 1.** Baseline Characterization of Opportunistic Screening and Nonopportunistic Group of the Study Cohort Without Imputation

Group	Opportunistic screening group (n = 2251)	Nonopportunistic group (n = 2983)	Total (N = 5234)	*P* value
Age, y				
<50	332 (14.7)	238 (8.0)	570 (10.9)	<.001
50-59	672 (29.9)	682 (22.9)	1354 (25.9)	
60-69	890 (39.5)	1307 (43.8)	2197 (42.0)	
≥70	357 (15.9)	756 (25.3)	1113 (21.3)	
Sex				
Female	1298 (57.7)	1220 (40.9)	2518 (48.1)	<.001
Male	953 (42.3)	1763 (59.1)	2716 (51.9)	
Ethnicity				
Han^a^	2240 (99.5)	2960 (99.2)	5200 (99.4)	.28
Non-Han	11 (0.5)	23 (0.8)	34 (0.7)	
Marital status				
Married	2237 (99.4)	2963 (99.3)	5200 (99.4)	>.99
Not married	14 (0.6)	18 (0.6)	32 (0.6)	
Missing	0	2 (<0.1)	2 (<0.1)	NA
Medical insurance				
Urban and rural residents basic medical insurance	864 (38.4)	1760 (59.0)	2624 (50.1)	<.001
Urban employees basic medical insurance	1254 (55.7)	959 (32.1)	2213 (42.3)	
Commercial insurance	112 (5.0)	208 (7.0)	320 (6.1)	
Free medical care	21 (0.9)	56 (1.9)	77 (1.5)	
Smoking				
Never	1690 (75.1)	1619 (54.3)	3309 (63.2)	<.001
Former	305 (13.5)	522 (17.5)	827 (15.8)	
Current	209 (9.3)	716 (24.0)	925 (17.7)	
Missing	47 (2.1)	126 (4.2)	173 (3.3)	NA
Alcohol use				
Yes	352 (15.6)	807 (27.1)	1159 (22.1)	<.001
Missing	154 (6.8)	383 (12.8)	537 (10.3)	NA
Family history of lung cancer				
Yes	264 (11.7)	267 (9.0)	531 (10.1)	.001
Missing	45 (2.0)	76 (2.5)	121 (2.3)	NA
TNM stage				
I	1729 (76.8)	770 (25.8)	2499 (47.7)	<.001
II	132 (5.9)	161 (5.4)	293 (5.6)	
III	216 (9.6)	565 (18.9)	781 (14.9)	
IV	133 (5.9)	1316 (44.1)	1449 (27.7)	
Missing	41 (1.8)	171 (5.7)	212 (4.1)	NA
Pathology				
Non–small cell lung cancer	2188 (97.2)	2515 (84.3)	4703 (89.9)	<.001
Small cell lung cancer	52 (2.3)	366 (12.3)	418 (8.0)	
Lung cancer NOS	11 (0.5)	102 (3.4)	113 (2.2)	
Tumor site				
Right upper lobe	743 (33.0)	792 (26.6)	1535 (29.3)	<.001
Left upper lobe	548 (24.3)	638 (21.4)	1186 (22.7)	
Right lower lobe	397 (18.1)	518 (17.4)	915 (17.5)	
Left lower lobe	353 (17.6)	468 (15.7)	821 (15.7)	
Right middle lobe	149 (6.6)	254 (8.5)	403 (7.7)	
Missing	61 (2.7)	313 (10.5)	374 (7.1)	NA
Surgery	1984 (88.1)	1009 (33.8)	2993 (57.2)	<.001
Radiotherapy	243 (10.8)	754 (25.3)	997 (19.0)	<.001
Chemotherapy	485 (21.5)	1318 (44.2)	1803 (34.4)	<.001
Targeted therapy	204 (9.1)	893 (29.9)	1097 (21.0)	<.001
Immunotherapy	33 (1.5)	144 (4.8)	177 (3.4)	<.001

Abbreviations: NA, not applicable; NOS, not otherwise specified.

^a^ The comparator ethnicities included Bai and Yao individuals.

In the opportunistic screening group, advanced lung cancer (stage III-IV) accounted for a much lower proportion (349 patients [15.79%]) than that in the nonopportunistic group (1881 patients [66.89%]) (*P* < .001). The proportion of patients who underwent surgical resection was higher in the opportunistic screening group (1984 patients [88.14%]) than in the nonopportunistic group (1009 patients [33.83%]). Moreover, the proportions of patients who underwent radiotherapy, chemotherapy, targeted therapy, or immunotherapy were lower in the opportunistic screening group compared with the nonopportunistic group (Table 1).

### Association of Opportunistic Screening With Lung Cancer and All-Cause Mortality

**Figure 3.**
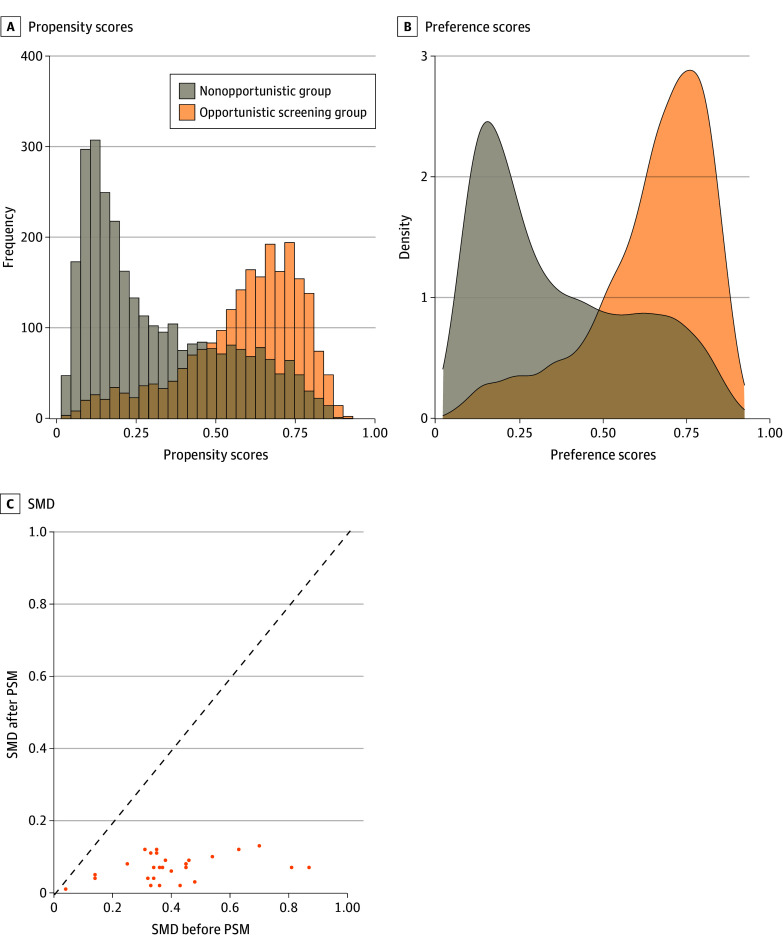
Distribution of Propensity Scores and Preference Scores of Opportunistic Screening and Nonopportunistic Groups and Absolute Values of Standardized Mean Differences (SMDs) of Covariables Before and After Propensity Score Matching (PSM)

A least absolute shrinkage and selection operator logistic regression model using 10-fold cross-validation was applied to filter out 28 variables (eg, age, smoking, medical insurance, respiratory diseases) that were then used to estimate propensity scores (eTable 6, eFigure 3 and eTable 7 in Supplement 1). The distribution of propensity scores and preference scores of the opportunistic screening and nonopportunistic groups are shown in Figure 3A and Figure 3B. After 1:1 matching according to PSM, 2728 patients (1364 in each group) were included in PSM analysis. The SMDs of 22 covariables after PSM were less than 0.10, except for carcinoembryonic antigen (SMD, 0.13), neutrophil count (SMD, 0.12), and the other 4 variables; therefore, we considered baseline characteristics of the 2 groups balanced and comparable (Figure 3C and eTable 6 in Supplement 1).

Based on the matched data, the univariate Cox regression model of opportunistic screening on lung cancer mortality and all-cause mortality was applied, respectively. Opportunistic screening was associated with a significant reduction in lung cancer–specific mortality (HR, 0.66; 95% CI, 0.55-0.80) and all-cause mortality (HR, 0.69; 95% CI, 0.58-0.83) after adjusting for lead time bias (Table 2). The length-bias correction factor for the specific lead time was 0.99 and 1.03 for lung cancer mortality and all-cause mortality, respectively (Table 2 and eTable 9 in Supplement 1). Opportunistic screening was associated with a significant reduction in lung cancer–specific mortality (HR, 0.66; 95% CI, 0.54-0.80) and all-cause mortality (HR, 0.72; 95% CI, 0.60-0.86) after adjusting for lead time and length bias (Table 2 and eFigure 4 in Supplement 1).

**Table 2.** Propensity Score Matching Analysis After Adjusting for Confounding, Lead Time Bias, and Length Bias

Lead time, d	HR (95%CI) after correction for confounder and lead time bias	Length-bias correction factor	HR (95%CI) after correction for confounder, lead time, and length biases
**Lung cancer death**			
210	0.66 (0.55-0.80)	0.993	0.66 (0.54-0.80)
150	0.62 (0.51-0.75)	1.001	0.62 (0.51-0.75)
180	0.64 (0.53-0.78)	1.026	0.66 (0.54-0.80)
240	0.69 (0.57-0.83)	1.038	0.71 (0.59-0.86)
270	0.71 (0.59-0.86)	1.051	0.75 (0.62-0.90)
**All-cause death**			
210	0.69 (0.58-0.83)	1.034	0.72 (0.60-0.86)
150	0.65 (0.54-0.78)	1.046	0.68 (0.57-0.82)
180	0.67 (0.56-0.81)	1.008	0.68 (0.57-0.81)
240	0.72 (0.60-0.86)	1.020	0.73 (0.61-0.88)
270	0.74 (0.62-0.89)	1.032	0.77 (0.64-0.92)

Abbreviation: HR, hazard ratio.

### Sensitivity Analysis

The results of propensity score analysis stratified by TNM stage are shown in eTable 8 in Supplement 1. The results of propensity score analysis, including propensity score regression adjustment and inverse probability treatment weighting after correcting possible lead time bias and length bias are shown in eTable 10 and eFigure 4 in Supplement 1. These findings were mostly consistent with those of the PSM method by adjusting the 6 variables with SMD exceeding 0.10 (eTable 10 and eFigure 4 in Supplement 1).

## Discussion

In this cohort study using a regional lung cancer cohort from Eastern China, we have assessed whether opportunistic LDCT screening was associated with improved prognosis of adults with lung cancer. Propensity score methods, including correction for both lead time and length biases, were fitted on lung cancer–specific mortality and all-cause mortality estimates to obtain robust conclusions. Our results highlight that opportunistic lung cancer screening with LDCT was associated with lower lung cancer mortality and all-cause mortality.

Data of patients with lung cancer in Weihai Municipal Hospital Healthcare Group were used to construct the lung cancer cohort. The Kaplan-Meier estimate of the 5-year survival rate of the lung cancer cohort was approximately 60%, which was much higher than the 5-year survival rate reported in the United States (25.4%).^2^ One potential reason for this difference is the difference in tumor stage composition ratio. In the US, stage I disease accounts for only 21% of lung cancer, while stage IV accounts for more than half (53%). However, in this study, stage I disease accounted for nearly half of all lung cancer (48%), and stage IV only accounted for 27%. Weihai City is located in the eastern coastal area of China, and its economic conditions are generally better than those of residents in the central and western areas of China. Meanwhile, hospitals in Weihai City actively and widely publicize the importance of early screening for cancer, and residents are likely more willing to accept health screening. As a result, the proportion of stage I diagnosed by opportunistic screening was as high as 78%, which was associated with lower mortality.

Other risk factors of lung cancer may differently impact mortality in Chinese and Western populations. In China, smoking prevalence is 3.5% among women and 49.7% among men, while smoking prevalence is 15.3% among women and 19.9% among men in the US.^34^ Among US patients with lung cancer, more than 84% of women and 90% of men have a history of smoking,^35^ and the proportion of cancer deaths attributable to smoking is nearly the same (80%) both in women and men.^36^ In our lung cancer cohort, nonsmokers accounted for 98% of female patients and 32% of male patients, and, as reported, only 18% of cancer deaths in women are attributable to smoking (75% in men) in China.^37^

People with a younger age and family history of lung cancer may be more likely to accept opportunistic LDCT screening. Furthermore, people with a family history of lung cancer may be more actively involved in opportunistic lung cancer screening because they may have a higher risk perception.^38^ Interestingly, we found that urban employees with medical insurance were more likely to receive a lung cancer diagnosis via opportunistic screening than those with medical insurance for urban and rural residents. The Regulations on Basic Medical Insurance for Urban Employees of China stipulate “Employers shall undertake relevant health examinations after employees participate in basic medical insurance,” which was consistent with our results. The proportion of smokers in the opportunistic screening group (24%) was lower than in the nonopportunistic group (45%), which was consistent with a 2022 study by Li et al.^15^ Smokers may have a fluke mentality (ie, because they have not gotten lung cancer yet, they will not) or they may have an avoidance mentality owing to fear of lung cancer and other diseases being detected lung cancer, and are reluctant to undergo LDCT examination.^39^

Our study has several strengths. To our knowledge, our study was the first to evaluate the association of opportunistic LDCT screening with prognosis among a large population of patients with lung cancer in China using propensity score methods. A retrospective study based on 2883 patients with lung cancer in Taiwan showed that screened cohorts had a higher proportion of women, younger age, and better prognosis compared with the nonscreened cohorts.^40^ However, the study by Wu et al^40^ had a smaller sample size, and it used Cox regression model controlling limited variables. Second, the quality of data in this study was high. Clinicians participated in and guided data cleaning and governance processes throughout by using the standardized data governance process of the Healthcare Big Data Research Institute at Shandong University, and supplementing information in the Shandong electronic case database. Third, in terms of data analysis, we used propensity score matching along with correction for lead time and length biases, and sensitivity analyses were carried out to ensure that our conclusions were robust. Estimations of lead time have been reported to show large variation, which could be attributed to the employed estimation methods, pathologies, stages, as well as diverse populations.^31,32,41^ To address this concern, several representative values of lead time were chosen from a reasonable range for the sensitivity analyses. To simultaneously address potential confounding and length biases, we combined the current approach to lead time with a correction factor derived from length bias correction. The survival benefit by opportunistic screening remained significant after adjustment for lead time and length biases.

### Limitations

This study has some limitations. First, the database did not collect information on patients’ nonparticipation in opportunistic screening and did not have information about reasons for nonparticipation. Second, the targeted therapy against the most common driver alterations, such as epidermal growth factor receptor (*EGFR*) and anaplastic lymphoma kinase (*ALK*) tyrosine kinase inhibitors, may also have an important impact on patient survival. *EGFR* alterations in patients with non–small cell lung cancer were more prevalent in, but not exclusive to, Asian females and nonsmokers.^42,43^ Third, in addition to screening aggressive tumors, opportunistic screening can also detect indolent tumors that may not cause clinical symptoms. The potential negative effects of screening, such as overdiagnosis, psychosocial effects (eg, anxiety), additional cost, morbidity, complications, and even mortality associated with cancer treatment,^44^ are also of concern. Current data are insufficient to support such studies. In the future, these issues can be explored in depth on the basis of the accumulation of relevant data.

## Conclusions

In this cohort study based on regional lung cancer cohort, we found that opportunistic screening with LDCT was significantly associated with lower lung cancer mortality and all-cause mortality in lung cancer population. Our findings suggest that opportunistic screening may serve as an important supplement to population screening to improve prognosis of adults with lung cancer.

## Notes

Supplement 1. **eMethods.** Correcting for Lead Time Bias and Length Bias **eTable 1.** Variable Coding of this Study **eTable 2.** The Eighth Edition TNM Stage Classification for Lung Cancer **eTable 3.** *ICD-10* Code of the Comorbidities **eTable 4.** The Quartile Cutoff Points for the Blood Indexes **eTable 5.** Baseline Characterization of Opportunistic Screening and Nonopportunistic Group of the Study Cohort **eTable 6.** Comparison of Baseline Characteristics of Opportunistic Screening Group and Nonopportunistic Group Before and After the Propensity Score Matching **eTable 7.** Variable Names From the Top to the Bottom in eFigure 3A **eTable 8.** Propensity Score Analysis Stratified by TNM Stage **eTable 9.** Results of HR for 2-year Case Fatality and Length-Bias Correction Factor Correcting for Lead Time and Length Biases **eTable 10.** Propensity Score Analysis After Correcting Possible Lead Time and Length Biases by Multiplying Length-Bias Correction Factor **eFigure 1.** Missing Rate of the Variables Selected in the Study Cohort **eFigure 2.** The Patterns of Missingness for the Top 10 Variables With the Most Missingness **eFigure 3.** Variable Selection Using the LASSO Logistic Regression Model **eFigure 4.** Forest Plot of Cox Regression Model Estimates for the Association Between Opportunistic LDCT Screening With Lung Cancer–Specific Death and All-Cause Death Using PSM, PSRA, and IPTW
eReferences

Supplement 2.
Retracted Article With Errors Highlighted

Supplement 3.
Replacement Article With Corrections Highlighted

Supplement 4.
Data Sharing Statement
