# Supplementary material for: Opportunistic Screening With Low-Dose Computed Tomography and Lung Cancer Mortality in China
Source: JAMA Netw Open. 2023 Dec 12;6(12):e2347176. doi: 10.1001/jamanetworkopen.2023.47176 (PMC10716726; doi:10.1001/jamanetworkopen.2023.47176)
Supplement: Supplement 1. — eMethods. Correcting for Lead Time Bias and Length Bias eTable 1. Variable Coding of this Study eTable 2. The Eighth Edition TNM Stage Classification for Lung Cancer eTable 3. ICD-10 Code of the Comorbidities eTable 4. The Quartile Cutoff Points for the Blood Indexes eTable 5. Baseline Characterization of Opportunistic Screening and Nonopportunistic Group of the Study Cohort eTable 6. Comparison of Baseline Characteristics of Opportunistic Screening Group and Nonopportunistic Group Before and After the Propensity Score Matching eTable 7. Variable Names From the Top to the Bottom in eFigure 3A eTable 8. Propensity Score Analysis Stratified by TNM Stage eTable 9. Results of HR for 2-year Case Fatality and Length-Bias Correction Factor Correcting for Lead Time and Length Biases eTable 10. Propensity Score Analysis After Correcting Possible Lead Time and Length Biases by Multiplying Length-Bias Correction Factor eFigure 1. Missing Rate of the Variables Selected in the Study Cohort eFigure 2. The Patterns of Missingness for the Top 10 Variables With the Most Missingness eFigure 3. Variable Selection Using the LASSO Logistic Regression Model eFigure 4. Forest Plot of Cox Regression Model Estimates for the Association Between Opportunistic LDCT Screening With Lung Cancer–Specific Death and All-Cause Death Using PSM, PSRA, and IPTW eReferences [file jamanetwopen-e2347176-s001.pdf]

Supplemental Online Content

Wang L, Qi Y, Liu A, et al. Opportunistic screening with low-dose computed tomography and lung cancer mortality in China. *JAMA Netw Open*. 2023;6(12):e2347176. doi:10.1001/jamanetworkopen.2023.47176

eMethods. Correcting for Lead Time Bias and Length Bias
eTable 1. Variable Coding of this Study
eTable 2. The Eighth Edition TNM Stage Classification for Lung Cancer
eTable 3. ICD-10 Code of the Comorbidities
eTable 4. The Quartile Cutoff Points for the Blood Indexes
eTable 5. Baseline Characterization of Opportunistic Screening and Nonopportunistic Group of the Study Cohort
eTable 6. Comparison of Baseline Characteristics of Opportunistic Screening Group and Nonopportunistic Group Before and After the Propensity Score Matching
eTable 7. Variable Names From the Top to the Bottom in eFigure 3A
eTable 8. Propensity Score Analysis Stratified by TNM Stage
eTable 9. Results of HR for 2-year Case Fatality and Length-Bias Correction Factor Correcting for Lead Time and Length Biases
eTable 10. Propensity Score Analysis After Correcting Possible Lead Time and Length Biases by Multiplying Length-Bias Correction Factor
eFigure 1. Missing Rate of the Variables Selected in the Study Cohort
eFigure 2. The Patterns of Missingness for the Top 10 Variables With the Most Missingness
eFigure 3. Variable Selection Using the LASSO Logistic Regression Model
eFigure 4. Forest Plot of Cox Regression Model Estimates for the Association Between Opportunistic LDCT Screening With Lung Cancer–Specific Death and All-Cause Death Using PSM, PSRA, and IPTW
eReferences

This supplemental material has been provided by the authors to give readers additional information about their work.

© 2023 Wang L et al. *JAMA Network Open.*

eMethods. Correcting for lead time bias and length bias

Lead Time Bias

According to studies reporting lead-time adjusted survival analysis[1–3], we excluded stage in the PSM model. Including this along with lead-time correction would likely obscure the effect of screening on mortality by overcorrecting benefits from stage shift. Variations in lead time have been reported in the literature.[4,5] Liu et al[4] reported lead times of 30 and 100 days. Yang et al[5] studied lead times within the range from 0.65 to 1.05 year. We therefore have focused on an intermediate lead time of 210 days and included 150, 180, 240, and 270 days for sensitivity analysis.

Based on the method proposed by Duffy et al.[6], the estimated expected lead time for each case in the screened group is $E(s) = \frac{1-e^{-\lambda t}}{\lambda}$ for a patient known to be alive at time *t* after diagnosis and $E(s) = \frac{1-e^{-\lambda t}-\lambda t e^{-\lambda t}}{\lambda(1-e^{-\lambda t})}$ for a patient known to be dead of lung cancer or all cause death at time *t* after diagnosis. By statistically averaging the $E(s)$ for all the patients in the screened group, one can obtain the corresponding median lead time. Following this procedure, we numerically determined the parameter λ for the lung cancer lead time of 150, 180, 210, 240, 270 days through Newton's bisection method. To be more specific, we iteratively updated the parameter λ to best produce the target lead time of 150, 180, 210, 240, 270 days. For lung cancer death, λ was 0.0066168, 0.0054577, 0.0046032, 0.0039346, 0.0033979 for the corresponding median lead time of 150, 180, 210, 240, 270 days; For all cause death, λ was 0.0066165, 0.0054547, 0.0045990, 0.0039324, 0.0033952 for the corresponding median lead time of 150, 180, 210, 240, 270 days.

Then, we use the determined λ to calculate the estimated expected lead time for each case in the screened group, $E(s) = \frac{1-e^{-\lambda t}}{\lambda}$ for a patient known to be alive at time *t* after diagnosis and $E(s) = \frac{1-e^{-\lambda t}-\lambda t e^{-\lambda t}}{\lambda(1-e^{-\lambda t})}$ for a patient known to be dead of lung cancer or all cause death at time *t* after diagnosis.

Third, a correction for lead time would involve subtracting $E(s)$ from the observed survival time for each case in the screened group.

Finally, we conducted the propensity score analysis to correct for both lead time bias and confounding bias.

Length Bias

For the correction for length bias, we first followed the Duffy et al's method[6]. p_1 was the observed 2-year case fatality (lung cancer fatality or all-cause fatality) for symptomatic lung cancers, p_2 was the observed 2-year case fatality (lung cancer fatality or all-cause fatality) for screen-detected lung cancers, and p_3 was the observed probability of screen detection. We calculated the corrected results for q ranging from

© 2023 Wang L et al. *JAMA Network Open.*

0.5-0.9 and θ ranging from 0.5-0.9. Then, a correction factor can be calculated by the ratio between the HR after correcting length bias and the HR without correction for length-bias. In order to address all 3 types of biases, the correction factor pertaining to length bias was multiplied by the calculated HR accouting for the corrections relating to lead time and confounding biases.

© 2023 Wang L et al. *JAMA Network Open.*

eTable 1. Variable coding of this study

Exposure factors	Variable type	Coding
Baseline characteristics		
Age (years)	Continuous	22-91
Sex	Categorical	1=Male; 2=Female
Ethnicity	Categorical	1=Han; 2=Non-Han
Marital status	Categorical	1=Married; 2=Non-married
Medical insurance	Categorical	1=Urban and rural residents basic medical insurance; 2=Urban employees basic medical insurance; 3=Commercial medical insurance; 4=Free medical care
Smoking	Categorical	1=Never; 2=Former; 3=Current
Drinking alcohol	Categorical	1=No; 2=Yes
Family history of lung cancer	Categorical	0=No; 1=Yes
Screening	Categorical	1=Opportunistic screening group; 2=Non-opportunistic group
Tumor characteristics		
TNM stage	Categorical	1=I; 2=II; 3=III; 4=IV
Pathology	Categorical	1=Non-small cell lung cancer; 2=Small cell lung cancer; 3=Lung cancer, NOS
Tumor location	Categorical	1=Right upper lobe; 2=Left upper lobe; 3=Right lower lobe; 4=Left lower lobe; 5=Right middle lobe
Comorbidities		
Respiratory diseases	Categorical	0=No; 1=Yes
Cardiovascular diseases	Categorical	0=No; 1=Yes
Digestive diseases	Categorical	0=No; 1=Yes
Chronic nephritis and kidney disease	Categorical	0=No; 1=Yes
Diabetes	Categorical	0=No; 1=Yes
Diffuse connective tissue disease	Categorical	0=No; 1=Yes
Anemia of chronic disease	Categorical	0=No; 1=Yes
Skin conditions	Categorical	0=No; 1=Yes
Baseline blood indexes		

© 2023 Wang L et al. *JAMA Network Open.*

Blood routine		
White blood cell count	Categorical	1=Q1; 2=Q2; 3=Q3; 4=Q4
Red blood cell count†	Categorical	1=Q1; 2=Q2; 3=Q3; 4=Q4
Hemoglobin amount†	Categorical	1=Q1; 2=Q2; 3=Q3; 4=Q4
Hematocrit†	Categorical	1=Q1; 2=Q2; 3=Q3; 4=Q4
Average red blood cell volume	Categorical	1=Q1; 2=Q2; 3=Q3; 4=Q4
Average amount of red blood cell hemoglobin	Categorical	1=Q1; 2=Q2; 3=Q3; 4=Q4
Average red blood cell hemoglobin concentration	Categorical	1=Q1; 2=Q2; 3=Q3; 4=Q4
Red blood cell distribution width CV	Categorical	1=Q1; 2=Q2; 3=Q3; 4=Q4
Red blood cell distribution width SD	Categorical	1=Q1; 2=Q2; 3=Q3; 4=Q4
Platelet count	Categorical	1=Q1; 2=Q2; 3=Q3; 4=Q4
Platelet hematocrit	Categorical	1=Q1; 2=Q2; 3=Q3; 4=Q4
Average platelet volume	Categorical	1=Q1; 2=Q2; 3=Q3; 4=Q4
Platelet volume distribution width	Categorical	1=Q1; 2=Q2; 3=Q3; 4=Q4
Lymphocyte count	Categorical	1=Q1; 2=Q2; 3=Q3; 4=Q4
Monocytes count	Categorical	1=Q1; 2=Q2; 3=Q3; 4=Q4
Neutrophil count	Categorical	1=Q1; 2=Q2; 3=Q3; 4=Q4
Eosinophil count	Categorical	1=Q1; 2=Q2; 3=Q3; 4=Q4
Basophil count	Categorical	1=Q1; 2=Q2; 3=Q3; 4=Q4
Percentage of lymphocytes	Categorical	1=Q1; 2=Q2; 3=Q3; 4=Q4
Monocytes ratio	Categorical	1=Q1; 2=Q2; 3=Q3; 4=Q4
Percentage of neutrophils	Categorical	1=Q1; 2=Q2; 3=Q3; 4=Q4
Percentage of eosinophils	Categorical	1=Q1; 2=Q2; 3=Q3; 4=Q4
Basophil percentage	Categorical	1=Q1; 2=Q2; 3=Q3; 4=Q4
Large platelet ratio	Categorical	1=Q1; 2=Q2; 3=Q3; 4=Q4
PLR	Categorical	1=Q1; 2=Q2; 3=Q3; 4=Q4
NLR	Categorical	1=Q1; 2=Q2; 3=Q3; 4=Q4
Low fluorescence intensity reticulocyte ratio	Categorical	1=Q1; 2=Q2; 3=Q3; 4=Q4
Medium fluorescence intensity reticulocyte ratio	Categorical	1=Q1; 2=Q2; 3=Q3; 4=Q4
High fluorescence intensity reticulocyte ratio	Categorical	1=Q1; 2=Q2; 3=Q3; 4=Q4
Percentage of reticulocytes	Categorical	1=Q1; 2=Q2; 3=Q3; 4=Q4
Reticulocyte count	Categorical	1=Q1; 2=Q2; 3=Q3; 4=Q4
Blood sugar		
Glucose	Categorical	1=Q1; 2=Q2; 3=Q3; 4=Q4

© 2023 Wang L et al. *JAMA Network Open.*

Coagulation indicators		
International standardized ratio	Categorical	1=Q1; 2=Q2; 3=Q3; 4=Q4
Prothrombin activity	Categorical	1=Q1; 2=Q2; 3=Q3; 4=Q4
Prothrombin time	Categorical	1=Q1; 2=Q2; 3=Q3; 4=Q4
Liver function		
Total protein	Categorical	1=Q1; 2=Q2; 3=Q3; 4=Q4
Albumin	Categorical	1=Q1; 2=Q2; 3=Q3; 4=Q4
Globulin	Categorical	1=Q1; 2=Q2; 3=Q3; 4=Q4
A/G	Categorical	1=Q1; 2=Q2; 3=Q3; 4=Q4
Total bilirubin	Categorical	1=Q1; 2=Q2; 3=Q3; 4=Q4
Direct bilirubin	Categorical	1=Q1; 2=Q2; 3=Q3; 4=Q4
Indirect bilirubin	Categorical	1=Q1; 2=Q2; 3=Q3; 4=Q4
Alanine aminotransferase†	Categorical	1=Q1; 2=Q2; 3=Q3; 4=Q4
Aspartate aminotransferase	Categorical	1=Q1; 2=Q2; 3=Q3; 4=Q4
ALT/AST	Categorical	1=Q1; 2=Q2; 3=Q3; 4=Q4
γ-glutamyl transpeptidase†	Categorical	1=Q1; 2=Q2; 3=Q3; 4=Q4
Alkaline phosphatase	Categorical	1=Q1; 2=Q2; 3=Q3; 4=Q4
Adenosine deaminase	Categorical	1=Q1; 2=Q2; 3=Q3; 4=Q4
Total bile acids	Categorical	1=Q1; 2=Q2; 3=Q3; 4=Q4
Kidney function		
Urea	Categorical	1=Q1; 2=Q2; 3=Q3; 4=Q4
Creatinine†	Categorical	1=Q1; 2=Q2; 3=Q3; 4=Q4
Urea nitrogen creatinine ratio	Categorical	1=Q1; 2=Q2; 3=Q3; 4=Q4
Uric acid†	Categorical	1=Q1; 2=Q2; 3=Q3; 4=Q4
Electrolyte		
Calcium	Categorical	1=Q1; 2=Q2; 3=Q3; 4=Q4
Potassium	Categorical	1=Q1; 2=Q2; 3=Q3; 4=Q4
Chlorine	Categorical	1=Q1; 2=Q2; 3=Q3; 4=Q4
Sodium	Categorical	1=Q1; 2=Q2; 3=Q3; 4=Q4
Tumor markers		
Carcinoembryonic antigen	Categorical	1=Q1; 2=Q2; 3=Q3; 4=Q4
Cytokeratin 19 fragments	Categorical	1=Q1; 2=Q2; 3=Q3; 4=Q4
Squamous cell carcinoma-associated antigen	Categorical	1=Q1; 2=Q2; 3=Q3; 4=Q4
Neuron-specific enolase	Categorical	1=Q1; 2=Q2; 3=Q3; 4=Q4
Cancer antigen 125	Categorical	1=Q1; 2=Q2; 3=Q3; 4=Q4
Treatment modalities		
Surgery	Categorical	0=No; 1=Yes
Radiotherapy	Categorical	0=No; 1=Yes

© 2023 Wang L et al. *JAMA Network Open.*

Chemotherapy	Categorical	0=No; 1=Yes
Immunotherapy	Categorical	0=No; 1=Yes
Targeted therapy	Categorical	0=No; 1=Yes

Q1, Q2, Q3, Q4 are quartiles; †: These variables are categorized into sex-disaggregated quartiles. The quartile cut off points are shown in eTable 4. PLR: platelet-to-lymphocyte ratio; NLR: neutrophil-to-lymphocyte ratio; A/G: albumin to globulin ratio; ALT/AST: ratio of alanine aminotransferase to aspartate aminotransferase; CA125: Carbohydrate antigen 125; Lung cancer NOS: The pathological type of lung cancer is not specifically specified

© 2023 Wang L et al. *JAMA Network Open.*

eTable 2. The Eighth Edition TNM stage classification for lung cancer

Group	TNM stage	Definition
T	TX	Primary tumor cannot be assessed, or tumor proven by the presence of malignant cells in sputum or bronchial washing but not visualized by imaging or bronchoscopy
	T1A	Tumor ≤1 cm in greatest dimension. Confined to the lung and visceral pleura, and does not involve the main bronchus; or tumor confined to the tube wall, regardless of size
	T1B	Tumor>1 cm but ≤2 cm in greatest dimension
	T1C	Tumor>2 cm but ≤3 cm in greatest dimension
	T2A	Tumor>3 cm but ≤4 cm or having any of the following features: involves the main bronchus, but without involvement of the carina; Invades visceral pleura; associated with atelectasis or obstructive pneumonitis that extends to the hilar region, involving part or all of the lung
	T2B	Tumor>4 cm but ≤5 cm in greatest dimension
	T3	Tumor>5 cm but ≤7 cm in greatest dimension or directly invading any of the following: chest wall, parietal pericardium; or separate tumor nodules in the same lobe as the primary
	T4	Tumor >7cm or invading viscera: heart, esophagus, trachea, mediastinum, diaphragm, protuberance or vertebral body; separate tumor nodules in an ipsilateral lobe different from that of the primary
N	NX	Regional lymph nodes cannot be assessed
	N0	No regional lymph node metastasis
	N1	Metastasis in ipsilateral peribronchial and/or ipsilateral hilar lymph nodes and intrapulmonary nodes, including involvement by direct extension
	N2	Metastasis in ipsilateral mediastinal and/or subcarinal lymph nodes
	N3	Metastasis in contralateral mediastinal, contralateral hilar, ipsilateral or contralateral scalene, or supraclavicular lymph nodes
M	MX	Distant metastasis cannot be assessed
	M0	No distant metastasis
	M1	Distant metastasis, such as pleural dissemination (malignant pleural effusion, pericardial effusion or pleural nodules); Satellite nodules appeared in the opposite lung lobe of the primary tumor with single metastasis; Distant metastasis (lung/extrapleural); Multiple metastatic foci

© 2023 Wang L et al. *JAMA Network Open.*

**Stage**	**N0**	**N1**	**N2**	**N3**
T1A	IA1	IIB	IIIA	IIIB
T1B	IA2	IIB	IIIA	IIIB
T1C	IA3	IIB	IIIA	IIIB
T2A	IB	IIB	IIIA	IIIB
T2B	IIA	IIB	IIIA	IIIB
T3	IIB	IIIA	IIIB	IIIC
T4	IIIA	IIIA	IIIB	IIIC
**M1**	IV	IV	IV	IV

© 2023 Wang L et al. *JAMA Network Open.*

eTable 3. ICD-10 code of the comorbidities

Disease	ICD-10 code
Respiratory diseases	A15-A16, J00-J06, J12-J18, J20-J21, J40-J44, J45-J46, J47, J60-J65, J80, J96, J841, J848, J849, G473
Cardiovascular diseases	I00-I09, I10-I15, I20-I51, I60-I69, I70, I71, I26, I80-I82
Digestive diseases	K21, K25-K29, K50-K51, K30, K58, B15-B19, K70, K760, K74, K92.2
Chronic nephritis and kidney disease	N02-N08, N11-N12, N14-N16, N18-N19
Diabetes	E10-E15
Diffuse connective tissue disease	M05-M08, M30-M36, M60
Anemia of chronic disease	D50-D53, D55-D59, D60-D61, D63-D64
Skin conditions	L20-L30, L40-L41

© 2023 Wang L et al. *JAMA Network Open.*

eTable 4. The quartile cut off points for the blood indexes

Blood indexes	Q1	Q2	Q3	Q4	Unit
Red blood cell count_male	[2.19, 4.1]	(4.1, 4.45]	(4.45, 4.78]	(4.78, 6.13]	*10^12/L
Red blood cell count_female	[2.42, 3.85]	(3.85, 4.15]	(4.15, 4.46]	(4.46, 6.13]	*10^12/L
Hemoglobin volume_male	[57, 126]	(126, 136]	(136, 147]	(147, 182]	g/L
Hemoglobin volume_female	[67, 114]	(114, 123]	(123, 132]	(132, 170]	g/L
Hematocrit_male	[20.7, 38.3]	(38.3, 41.2]	(41.2, 44.1]	(44.1, 56.5]	%
Hematocrit_female	[22.6, 35]	(35, 37.6]	(37.6, 40.2]	(40.2, 52.9]	%
Alanine aminotransferase_male	[0.9, 10.5]	(10.5, 14.3]	(14.3, 20.9]	(20.9, 203]	U/L
Alanine aminotransferase_female	[1.5, 9.5]	(9.5, 12.5]	(12.5, 17.4]	(17.4, 203]	U/L
Gamma-glutamyl transpeptidase_male	[3.5, 17]	(17, 24]	(24, 37]	(37, 202]	U/L
Gamma-glutamyl transpeptidase_female	[0.1, 11.2]	(11.2, 15.2]	(15.2, 22.2]	(22.2, 202]	U/L
Creatinine_male	[22, 58]	(58, 66]	(66, 75.3]	(75.3, 154]	umol/L
Creatinine_female	[23, 46.6]	(46.6, 53]	(53, 61]	(61, 154]	umol/L
Uric acid_male	[41.8, 220]	(220, 276]	(276, 337]	(337, 626]	umol/L
Uric acid_female	[41.8, 169]	(169, 216]	(216, 268]	(268, 626]	umol/L
White blood cell count	[1.21, 6.42]	(6.42, 8.18]	(8.18, 10.5]	(10.5, 20]	*10^9/L
Large platelet ratio	[6.3, 17.9]	(17.9, 22.4]	(22.4, 28.1]	(28.1, 58.8]	%
Monocytes ratio	[0.7, 5.5]	(5.5, 6.6]	(6.6, 7.8]	(7.8, 16.5]	%
Monocytes count	[0.04, 0.4]	(0.4, 0.54]	(0.54, 0.72]	(0.72, 2.03]	*10^9/L
Low fluorescence intensity reticulocyte ratio	[69.7, 91.1]	(91.1, 94.2]	(94.2, 96.7]	(96.7, 100]	%
Medium fluorescence intensity reticulocyte ratio	[0, 3.2]	(3.2, 5.6]	(5.6, 8.4]	(8.4, 20.3]	%

© 2023 Wang L et al. *JAMA Network Open.*

High fluorescence intensity reticulocyte ratio	0	(0, 0.4]	(0.4, 9.1]	-	%
Red blood cell distribution width CV	[10.7, 12.4]	(12.4, 12.8]	(12.8, 13.3]	(13.3, 20.1]	%
Red blood cell distribution width SD	[33.9, 40.2]	(40.2, 41.8]	(41.8, 43.8]	(43.8, 60.1]	fL
Percentage of lymphocytes	[2.72, 12.7]	(12.7, 19.3]	(19.3, 26.9]	(26.9, 58.9]	%
Lymphocyte count	[0.18, 1.15]	(1.15, 1.52]	(1.52, 1.96]	(1.96, 5.08]	*10^9/L
Average red blood cell volume	[58.9, 88.9]	(88.9, 91.9]	(91.9, 94.8]	(94.8, 112]	fL
Average amount of red blood cell hemoglobin	[16.8, 29.2]	(29.2, 30.3]	(30.3, 31.3]	(31.3, 39.6]	pg
Average red blood cell hemoglobin concentration	[279, 322]	(322, 329]	(329, 335]	(335, 366]	g/L
Average platelet volume	[6.6, 8.9]	(8.9, 9.6]	(9.6, 10.4]	(10.4, 14.2]	fL
Basophil percentage	[0, 0.1]	(0.1, 0.2]	(0.2, 0.4]	(0.4, 1.51]	%
Basophil count	[0, 0.01]	(0.01, 0.02]	(0.02, 0.03]	(0.03, 0.12]	*10^9/L
Percentage of eosinophils	[0, 0.3]	(0.3, 0.9]	(0.9, 1.9]	(1.9, 12.1]	%
Eosinophil count	[0, 0.03]	(0.03, 0.07]	(0.07, 0.14]	(0.14, 1.56]	*10^9/L
Percentage of reticulocytes	[0.3, 0.9]	(0.9, 1.2]	(1.2, 1.5]	(1.5, 3.1]	%
Reticulocyte count	[0.011, 0.04]	(0.04, 0.05]	(0.05, 0.062]	(0.062, 0.154]	*10^12/L
Platelet count	[46, 187]	(187, 226]	(226, 273]	(273, 609]	*10^9/L
Platelet volume distribution width	[7.5, 11.6]	(11.6, 15.6]	(15.6, 16.1]	(16.1, 23.1]	fL
Platelet hematocrit	[0.05, 0.18]	(0.18, 0.22]	(0.22, 0.26]	(0.26, 0.51]	mL/L
Percentage of neutrophils	[28.4, 63.7]	(63.7, 71.8]	(71.8, 79.5]	(79.5, 95.2]	%
Neutrophil count	[0.32, 4.15]	(4.15, 5.83]	(5.83, 8.14]	(8.14, 24.1]	*10^9/L
PLR	[13.6, 115]	(115, 150]	(150, 199]	(199, 612]	−
NLR	[0.12, 2.38]	(2.38, 3.72]	(3.72, 6.2]	(6.2, 25.6]	−
Urea	[1, 4.2]	(4.2, 5.2]	(5.2, 6.4]	(6.4, 20.3]	mmol/l

© 2023 Wang L et al. *JAMA Network Open.*

Urea nitrogen creatinine ratio	[0.023, 0.069]	(0.069, 0.086]	(0.086, 0.107]	(0.107, 0.258]	−
ALT/AST	[0.05, 0.64]	(0.64, 0.83]	(0.83, 1.11]	(1.11, 3.83]	−
Albumin	[18.8, 35.3]	(35.3, 38.4]	(38.4, 42]	(42, 63.4]	g/l
A/G	[0.49, 1.22]	(1.22, 1.4]	(1.4, 1.59]	(1.59, 3.01]	−
Indirect bilirubin	[0.2, 4.8]	(4.8, 6.9]	(6.9, 9.9]	(9.9, 40.3]	umol/L
Alkaline phosphatase	[11, 53.7]	(53.7, 66]	(66, 83]	(83, 305]	U/L
Globulin	[12.8, 24.8]	(24.8, 27.7]	(27.7, 31.2]	(31.2, 58.5]	g/L
Aspartate aminotransferase	[4.2, 13.3]	(13.3, 16.2]	(16.2, 20.7]	(20.7, 182]	U/L
Adenosine deaminase	[0, 6.7]	(6.7, 9]	(9, 12]	(12, 30.2]	U/L
Direct bilirubin	[0.4, 3.6]	(3.6, 4.6]	(4.6, 6.1]	(6.1, 42.7]	umol/L
Total bilirubin	[1.8, 8.6]	(8.6, 11.6]	(11.6, 15.9]	(15.9, 61.4]	umol/L
Total bile acids	[0, 1.2]	(1.2, 2.2]	(2.2, 3.9]	(3.9, 25.5]	umol/L
Total protein	[39.7, 61.8]	(61.8, 66.4]	(66.4, 72]	(72, 97.1]	g/L
Tumor markers	[0, 1.73]	(1.73, 2.91]	(2.91, 6.66]	(6.66, 60.2]	ng/ml
Carcinoembryonic antigen	[0.1, 1.8]	(1.8, 2.9]	(2.9, 4.9]	(4.9, 60.9]	ng/ml
Cytokeratin 19 fragments	[0, 0.4]	(0.4, 0.72]	(0.72, 1.11]	(1.11, 43.6]	ng/ml
Squamous cell carcinoma-associated antigen	[0.6, 10.8]	(10.8, 12.7]	(12.7, 15.8]	(15.8, 100]	ng/ml
Neuron-specific enolase	[0.1, 8.89]	(8.89, 13.3]	(13.3, 26.5]	(26.5, 200]	U/ml
Glucose	[2.21, 4.91]	(4.91, 5.44]	(5.44, 6.17]	(6.17, 18.5]	mmol/l
Calcium	[1.79, 2.08]	(2.08, 2.17]	(2.17, 2.28]	(2.28, 2.77]	mmol/L
Potassium	[2.55, 3.76]	(3.76, 4.03]	(4.03, 4.32]	(4.32, 5.97]	mmol/L
Chlorine	[79.2, 101]	(101, 103]	(103, 106]	(106, 118]	mmol/l
Sodium	[125, 139]	(139, 141]	(141, 143]	(143, 151]	mmol/L
International standardized ratio	[0.76, 0.97]	(0.97, 1.01]	(1.01, 1.05]	(1.05, 1.52]	−
Prothrombin activity	[59, 88]	(88, 95]	(95, 101]	(101, 126]	%
Prothrombin time	[9.3, 11.8]	(11.8, 12.2]	(12.2, 12.7]	(12.7, 20.6]	sec

PLR: Platelet to lymphocyte ratio; NLR: neutrophils to lymphocytes ratio; A/G: albumin to globulin ratio; ALT/AST: ratio of alanine aminotransferase to aspartate aminotransferase; CA125: carbohydrate antigen 125

eTable 5. Baseline characterization (all the comorbidity and blood indexes) of opportunistic screening and non-opportunistic group of the study cohort

Variable	Group	Opportunistic screening group N=2251	Non-opportunistic group N=2983	Total N=5234	*P*
Respiratory diseases	No	1,574 (69.92)	1,609 (53.94)	3,183 (60.81)	<0.001*
	Yes	677 (30.08)	1,374 (46.06)	2,051 (39.19)	
Cardiovascular diseases	No	1,355 (60.20)	1,540 (51.63)	2,895 (55.31)	<0.001*
	Yes	896 (39.80)	1,443 (48.37)	2,339 (44.69)	
Digestive diseases	No	1,873 (83.21)	2,497 (83.71)	4,370 (83.49)	0.656
	Yes	378 (16.79)	486 (16.29)	864 (16.51)	
Chronic nephritis and kidney disease	No	2,201 (97.78)	2,905 (97.39)	5,106 (97.55)	0.411
	Yes	50 (2.22)	78 (2.61)	128 (2.45)	
Diabetes	No	1,936 (86.01)	2,607 (87.40)	4,543 (86.80)	0.153
	Yes	315 (13.99)	376 (12.60)	691 (13.20)	
Anemia of chronic disease	No	2,220 (98.62)	2,870 (96.21)	5,090 (97.25)	<0.001*
	Yes	31 (1.38)	113 (3.79)	144 (2.75)	
Diffuse connective tissue disease	No	2,220 (98.62)	2,928 (98.16)	5,148 (98.36)	0.228
	Yes	31 (1.38)	55 (1.84)	86 (1.64)	
Skin conditions	No	2,187 (97.16)	2,927 (98.12)	5,114 (97.71)	0.027*
	Yes	64 (2.84)	56 (1.88)	120 (2.29)	
White blood cell count	Q1	477 (21.47)	801 (28.16)	1,278 (25.23)	<0.001*
	Q2	474 (21.33)	785 (27.60)	1,259 (24.85)	
	Q3	607 (27.32)	660 (23.21)	1,267 (25.01)	
	Q4	664 (29.88)	598 (21.03)	1,262 (24.91)	
Large platelet ratio	Q1	396 (17.87)	878 (30.97)	1,274 (25.22)	<0.001*
	Q2	582 (26.26)	685 (24.16)	1,267 (25.08)	
	Q3	570 (25.72)	680 (23.99)	1,250 (24.75)	
	Q4	668 (30.14)	592 (20.88)	1,260 (24.95)	
Monocytes ratio	Q1	606 (27.27)	703 (24.72)	1,309 (25.84)	<0.001*

© 2023 Wang L et al. *JAMA Network Open.*

	Q2	586 (26.37)	638 (22.43)	1,224 (24.16)	
	Q3	532 (23.94)	736 (25.88)	1,268 (25.03)	
	Q4	498 (22.41)	767 (26.97)	1,265 (24.97)	
Monocytes count	Q1	546 (24.57)	790 (27.78)	1,336 (26.37)	<0.001*
	Q2	492 (22.14)	764 (26.86)	1,256 (24.79)	
	Q3	569 (25.61)	658 (23.14)	1,227 (24.22)	
	Q4	615 (27.68)	632 (22.22)	1,247 (24.62)	
Low fluorescence intensity reticulocyte ratio	Q1	417 (18.89)	784 (30.40)	1,201 (25.09)	<0.001*
	Q2	615 (27.87)	614 (23.81)	1,229 (25.68)	
	Q3	571 (25.87)	609 (23.61)	1,180 (24.66)	
	Q4	604 (27.37)	572 (22.18)	1,176 (24.57)	
High fluorescence intensity reticulocyte ratio	Q1	1,486 (67.33)	1,351 (52.38)	2,837 (59.28)	<0.001*
	Q2	360 (16.31)	467 (18.11)	827 (17.28)	
	Q3	361 (16.36)	761 (29.51)	1,122 (23.44)	
Hematocrit	Q1	587 (26.36)	700 (24.57)	1,287 (25.35)	<0.001*
	Q2	633 (28.42)	640 (22.46)	1,273 (25.08)	
	Q3	554 (24.88)	714 (25.06)	1,268 (24.98)	
	Q4	453 (20.34)	795 (27.90)	1,248 (24.59)	
Red blood cell distribution width CV	Q1	613 (27.59)	749 (26.34)	1,362 (26.89)	<0.001*
	Q2	615 (27.68)	662 (23.28)	1,277 (25.21)	
	Q3	521 (23.45)	704 (24.75)	1,225 (24.18)	
	Q4	473 (21.29)	729 (25.63)	1,202 (23.73)	
Red blood cell distribution width SD	Q1	564 (25.38)	729 (25.63)	1,293 (25.52)	0.002*
	Q2	589 (26.51)	673 (23.66)	1,262 (24.91)	
	Q3	588 (26.46)	704 (24.75)	1,292 (25.50)	
	Q4	481 (21.65)	738 (25.95)	1,219 (24.06)	
Red blood cell count	Q1	632 (28.44)	663 (23.31)	1,295 (25.56)	<0.001*
	Q2	612 (27.54)	649 (22.82)	1,261 (24.89)	
	Q3	542 (24.39)	718 (25.25)	1,260 (24.87)	
	Q4	436 (19.62)	814 (28.62)	1,250 (24.67)	
Percentage of lymphocytes	Q1	727 (32.72)	542 (19.06)	1,269 (25.05)	<0.001*

© 2023 Wang L et al. *JAMA Network Open.*

	Q2	584 (26.28)	681 (23.95)	1,265 (24.97)	
	Q3	467 (21.02)	800 (28.13)	1,267 (25.01)	
	Q4	444 (19.98)	821 (28.87)	1,265 (24.97)	
Lymphocyte count	Q1	668 (30.06)	605 (21.27)	1,273 (25.13)	<0.001*
	Q2	584 (26.28)	706 (24.82)	1,290 (25.46)	
	Q3	510 (22.95)	752 (26.44)	1,262 (24.91)	
	Q4	460 (20.70)	781 (27.46)	1,241 (24.50)	
Average red blood cell volume	Q1	466 (21.76)	765 (27.70)	1,231 (25.10)	<0.001*
	Q2	556 (25.96)	702 (25.42)	1,258 (25.65)	
	Q3	552 (25.77)	652 (23.61)	1,204 (24.55)	
	Q4	568 (26.52)	643 (23.28)	1,211 (24.69)	
Average amount of red blood cell hemoglobin	Q1	512 (23.04)	787 (27.67)	1,299 (25.64)	0.001*
	Q2	621 (27.95)	721 (25.35)	1,342 (26.49)	
	Q3	543 (24.44)	632 (22.22)	1,175 (23.19)	
	Q4	546 (24.57)	704 (24.75)	1,250 (24.67)	
Average red blood cell hemoglobin concentration	Q1	583 (26.19)	738 (25.92)	1,321 (26.04)	<0.001*
	Q2	620 (27.85)	716 (25.15)	1,336 (26.34)	
	Q3	554 (24.89)	622 (21.85)	1,176 (23.18)	
	Q4	469 (21.07)	771 (27.08)	1,240 (24.44)	
Average platelet volume	Q1	453 (20.44)	896 (31.60)	1,349 (26.71)	<0.001*
	Q2	568 (25.63)	669 (23.60)	1,237 (24.49)	
	Q3	582 (26.26)	709 (25.01)	1,291 (25.56)	
	Q4	613 (27.66)	561 (19.79)	1,174 (23.24)	
Basophil percentage	Q1	1,081 (48.65)	1,010 (35.51)	2,091 (41.28)	<0.001*
	Q2	479 (21.56)	455 (16.00)	934 (18.44)	
	Q3	446 (20.07)	695 (24.44)	1,141 (22.52)	
	Q4	216 (9.72)	684 (24.05)	900 (17.77)	
Basophil count	Q1	1,190 (53.56)	1,228 (43.18)	2,418 (47.73)	<0.001*
	Q2	539 (24.26)	526 (18.50)	1,065 (21.02)	
	Q3	283 (12.74)	447 (15.72)	730 (14.41)	
	Q4	210 (9.45)	643 (22.61)	853 (16.84)	
Percentage of	Q1	777 (34.97)	580 (20.39)	1,357 (26.79)	<0.001*

© 2023 Wang L et al. *JAMA Network Open.*

eosinophils					
	Q2	614 (27.63)	574 (20.18)	1,188 (23.45)	
	Q3	464 (20.88)	799 (28.09)	1,263 (24.93)	
	Q4	367 (16.52)	891 (31.33)	1,258 (24.83)	
Eosinophil count	Q1	842 (37.89)	665 (23.38)	1,507 (29.75)	<0.001*
	Q2	564 (25.38)	553 (19.44)	1,117 (22.05)	
	Q3	446 (20.07)	777 (27.32)	1,223 (24.14)	
	Q4	370 (16.65)	849 (29.85)	1,219 (24.06)	
Percentage of reticulocytes	Q1	585 (26.51)	612 (23.73)	1,197 (25.01)	0.002*
	Q2	738 (33.44)	810 (31.41)	1,548 (32.34)	
	Q3	468 (21.21)	567 (21.99)	1,035 (21.63)	
	Q4	416 (18.85)	590 (22.88)	1,006 (21.02)	
Reticulocyte count	Q1	638 (28.91)	619 (24.01)	1,257 (26.27)	<0.001*
	Q2	578 (26.19)	570 (22.11)	1,148 (23.99)	
	Q3	558 (25.28)	687 (26.65)	1,245 (26.02)	
	Q4	433 (19.62)	702 (27.23)	1,135 (23.72)	
Hemoglobin amount	Q1	609 (27.35)	723 (25.37)	1,332 (26.24)	<0.001*
	Q2	616 (27.66)	651 (22.84)	1,267 (24.96)	
	Q3	562 (25.24)	709 (24.88)	1,271 (25.03)	
	Q4	440 (19.76)	767 (26.91)	1,207 (23.77)	
Platelet count	Q1	718 (32.31)	571 (20.08)	1,289 (25.44)	<0.001*
	Q2	640 (28.80)	608 (21.38)	1,248 (24.63)	
	Q3	549 (24.71)	713 (25.07)	1,262 (24.91)	
	Q4	315 (14.18)	952 (33.47)	1,267 (25.01)	
Platelet volume distribution width	Q1	417 (18.82)	854 (30.12)	1,271 (25.16)	<0.001*
	Q2	528 (23.83)	750 (26.46)	1,278 (25.30)	
	Q3	746 (33.66)	797 (28.11)	1,543 (30.55)	
	Q4	525 (23.69)	434 (15.31)	959 (18.99)	
Platelet hematocrit	Q1	748 (33.75)	613 (21.62)	1,361 (26.95)	<0.001*
	Q2	659 (29.74)	730 (25.75)	1,389 (27.50)	
	Q3	481 (21.71)	651 (22.96)	1,132 (22.41)	
	Q4	328 (14.80)	841 (29.66)	1,169 (23.14)	
Percentage of neutrophils	Q1	420 (18.90)	862 (30.31)	1,282 (25.31)	<0.001*
	Q2	463 (20.84)	796 (27.99)	1,259 (24.85)	
	Q3	594 (26.73)	669 (23.52)	1,263 (24.93)	

© 2023 Wang L et al. *JAMA Network Open.*

	Q4	745 (33.53)	517 (18.18)	1,262 (24.91)	
Neutrophil count	Q1	447 (20.12)	824 (28.97)	1,271 (25.09)	<0.001*
	Q2	469 (21.11)	800 (28.13)	1,269 (25.05)	
	Q3	615 (27.68)	648 (22.78)	1,263 (24.93)	
	Q4	691 (31.10)	572 (20.11)	1,263 (24.93)	
Reticulocyte count	Q1	629 (28.50)	601 (23.30)	1,230 (25.70)	<0.001*
	Q2	569 (25.78)	604 (23.42)	1,173 (24.51)	
	Q3	594 (26.91)	604 (23.42)	1,198 (25.03)	
	Q4	415 (18.80)	770 (29.86)	1,185 (24.76)	
PLR	Q1	590 (26.55)	677 (23.80)	1,267 (25.01)	0.009*
	Q2	569 (25.61)	699 (24.58)	1,268 (25.03)	
	Q3	559 (25.16)	714 (25.11)	1,273 (25.13)	
	Q4	504 (22.68)	754 (26.51)	1,258 (24.83)	
NLR	Q1	436 (19.62)	837 (29.43)	1,273 (25.13)	<0.001*
	Q2	464 (20.88)	799 (28.09)	1,263 (24.93)	
	Q3	593 (26.69)	674 (23.70)	1,267 (25.01)	
	Q4	729 (32.81)	534 (18.78)	1,263 (24.93)	
Creatinine	Q1	489 (22.27)	798 (28.06)	1,287 (25.54)	<0.001*
	Q2	568 (25.87)	705 (24.79)	1,273 (25.26)	
	Q3	587 (26.73)	668 (23.49)	1,255 (24.90)	
	Q4	552 (25.14)	673 (23.66)	1,225 (24.31)	
Urea	Q1	530 (24.15)	812 (28.62)	1,342 (26.67)	<0.001*
	Q2	508 (23.14)	725 (25.56)	1,233 (24.50)	
	Q3	553 (25.19)	646 (22.77)	1,199 (23.83)	
	Q4	604 (27.52)	654 (23.05)	1,258 (25.00)	
Urea nitrogen creatinine ratio	Q1	493 (22.46)	820 (28.91)	1,313 (26.10)	<0.001*
	Q2	517 (23.55)	744 (26.23)	1,261 (25.06)	
	Q3	561 (25.56)	671 (23.66)	1,232 (24.49)	
	Q4	624 (28.43)	601 (21.19)	1,225 (24.35)	
Uric acid	Q1	607 (27.82)	627 (22.79)	1,234 (25.02)	<0.001*
	Q2	590 (27.04)	651 (23.66)	1,241 (25.16)	
	Q3	524 (24.01)	703 (25.55)	1,227 (24.87)	
	Q4	461 (21.13)	770 (27.99)	1,231 (24.95)	
ALT/AST	Q1	559 (25.33)	703 (24.82)	1,262 (25.04)	<0.001*
	Q2	603 (27.32)	673 (23.76)	1,276 (25.32)	
	Q3	563 (25.51)	692 (24.44)	1,255 (24.91)	
	Q4	482 (21.84)	764 (26.98)	1,246 (24.73)	
γ-glutamyl transpeptidase	Q1	683 (30.98)	568 (20.35)	1,251 (25.04)	<0.001*

© 2023 Wang L et al. *JAMA Network Open.*

	Q2	593 (26.89)	672 (24.08)	1,265 (25.32)	
	Q3	504 (22.86)	740 (26.51)	1,244 (24.90)	
	Q4	425 (19.27)	811 (29.06)	1,236 (24.74)	
Albumin	Q1	541 (24.52)	735 (26.27)	1,276 (25.50)	<0.001*
	Q2	633 (28.69)	613 (21.91)	1,246 (24.90)	
	Q3	535 (24.25)	712 (25.45)	1,247 (24.92)	
	Q4	497 (22.53)	738 (26.38)	1,235 (24.68)	
A/G	Q1	340 (15.42)	909 (32.62)	1,249 (25.02)	<0.001*
	Q2	598 (27.12)	696 (24.97)	1,294 (25.92)	
	Q3	656 (29.75)	597 (21.42)	1,253 (25.10)	
	Q4	611 (27.71)	585 (20.99)	1,196 (23.96)	
Alanine aminotransferase	Q1	526 (23.82)	753 (26.56)	1,279 (25.36)	0.002*
	Q2	572 (25.91)	676 (23.84)	1,248 (24.75)	
	Q3	592 (26.81)	667 (23.53)	1,259 (24.97)	
	Q4	518 (23.46)	739 (26.07)	1,257 (24.93)	
Indirect bilirubin	Q1	406 (18.40)	847 (30.68)	1,253 (25.23)	<0.001*
	Q2	557 (25.25)	680 (24.63)	1,237 (24.90)	
	Q3	589 (26.70)	655 (23.72)	1,244 (25.05)	
	Q4	654 (29.65)	579 (20.97)	1,233 (24.82)	
Alkaline phosphatase	Q1	808 (36.95)	426 (15.91)	1,234 (25.37)	<0.001*
	Q2	600 (27.43)	602 (22.49)	1,202 (24.71)	
	Q3	484 (22.13)	728 (27.19)	1,212 (24.92)	
	Q4	295 (13.49)	921 (34.40)	1,216 (25.00)	
Globulin	Q1	715 (32.43)	561 (20.13)	1,276 (25.56)	<0.001*
	Q2	651 (29.52)	610 (21.89)	1,261 (25.26)	
	Q3	525 (23.81)	690 (24.76)	1,215 (24.34)	
	Q4	314 (14.24)	926 (33.23)	1,240 (24.84)	
Aspartate aminotransferase	Q1	487 (22.06)	791 (27.77)	1,278 (25.28)	<0.001*
	Q2	588 (26.63)	668 (23.46)	1,256 (24.84)	
	Q3	599 (27.13)	664 (23.31)	1,263 (24.98)	
	Q4	534 (24.18)	725 (25.46)	1,259 (24.90)	
Adenosine deaminase	Q1	611 (29.67)	454 (20.60)	1,065 (24.98)	<0.001*
	Q2	563 (27.34)	520 (23.59)	1,083 (25.40)	
	Q3	452 (21.95)	597 (27.09)	1,049 (24.61)	
	Q4	433 (21.03)	633 (28.72)	1,066 (25.01)	
Direct bilirubin	Q1	403 (18.27)	850 (30.77)	1,253 (25.22)	<0.001*
	Q2	542 (24.57)	727 (26.32)	1,269 (25.54)	

© 2023 Wang L et al. *JAMA Network Open.*

	Q3	602 (27.29)	641 (23.21)	1,243 (25.02)	
	Q4	659 (29.87)	544 (19.70)	1,203 (24.21)	
Total bilirubin	Q1	416 (18.86)	853 (30.87)	1,269 (25.54)	<0.001*
	Q2	535 (24.25)	712 (25.77)	1,247 (25.10)	
	Q3	582 (26.38)	637 (23.05)	1,219 (24.53)	
	Q4	673 (30.51)	561 (20.30)	1,234 (24.83)	
Total bile acids	Q1	686 (32.45)	454 (19.20)	1,140 (25.45)	<0.001*
	Q2	560 (26.49)	576 (24.36)	1,136 (25.36)	
	Q3	473 (22.37)	652 (27.57)	1,125 (25.12)	
	Q4	395 (18.68)	683 (28.88)	1,078 (24.07)	
Total protein	Q1	684 (31.02)	577 (20.70)	1,261 (25.26)	<0.001*
	Q2	645 (29.25)	595 (21.35)	1,240 (24.84)	
	Q3	479 (21.72)	778 (27.92)	1,257 (25.18)	
	Q4	397 (18.00)	837 (30.03)	1,234 (24.72)	
Glucose	Q1	575 (26.03)	696 (24.58)	1,271 (25.22)	0.334
	Q2	571 (25.85)	699 (24.69)	1,270 (25.20)	
	Q3	529 (23.95)	711 (25.11)	1,240 (24.60)	
	Q4	534 (24.17)	725 (25.61)	1,259 (24.98)	
Calcium	Q1	697 (31.78)	558 (19.86)	1,255 (25.09)	<0.001*
	Q2	646 (29.46)	705 (25.10)	1,351 (27.01)	
	Q3	460 (20.98)	685 (24.39)	1,145 (22.89)	
	Q4	390 (17.78)	861 (30.65)	1,251 (25.01)	
Potassium	Q1	686 (31.15)	600 (21.04)	1,286 (25.45)	<0.001*
	Q2	622 (28.25)	658 (23.07)	1,280 (25.33)	
	Q3	478 (21.71)	745 (26.12)	1,223 (24.20)	
	Q4	416 (18.89)	849 (29.77)	1,265 (25.03)	
Chlorine	Q1	470 (21.43)	982 (34.57)	1,452 (28.84)	<0.001*
	Q2	423 (19.29)	660 (23.23)	1,083 (21.51)	
	Q3	693 (31.60)	728 (25.62)	1,421 (28.23)	
	Q4	607 (27.68)	471 (16.58)	1,078 (21.41)	
Sodium	Q1	414 (18.86)	850 (29.89)	1,264 (25.08)	<0.001*
	Q2	710 (32.35)	925 (32.52)	1,635 (32.45)	
	Q3	575 (26.20)	608 (21.38)	1,183 (23.48)	
	Q4	496 (22.60)	461 (16.21)	957 (18.99)	
International standardized ratio	Q1	752 (37.21)	661 (24.12)	1,413 (29.67)	<0.001*
	Q2	569 (28.15)	613 (22.36)	1,182 (24.82)	
	Q3	488 (24.15)	668 (24.37)	1,156 (24.28)	
	Q4	212 (10.49)	799 (29.15)	1,011 (21.23)	
Prothrombin activity	Q1	293 (14.71)	849 (33.25)	1,142 (25.13)	<0.001*

© 2023 Wang L et al. *JAMA Network Open.*

	Q2	635 (31.88)	705 (27.61)	1,340 (29.48)	
	Q3	566 (28.41)	504 (19.74)	1,070 (23.54)	
	Q4	498 (25.00)	495 (19.39)	993 (21.85)	
Prothrombin time	Q1	751 (37.16)	594 (21.67)	1,345 (28.24)	<0.001*
	Q2	605 (29.94)	581 (21.20)	1,186 (24.91)	
	Q3	418 (20.68)	632 (23.06)	1,050 (22.05)	
	Q4	247 (12.22)	934 (34.08)	1,181 (24.80)	
Carcinoembryonic antigen	Q1	768 (36.26)	438 (16.29)	1,206 (25.09)	<0.001*
	Q2	651 (30.74)	548 (20.39)	1,199 (24.95)	
	Q3	466 (22.00)	732 (27.23)	1,198 (24.93)	
	Q4	233 (11.00)	970 (36.09)	1,203 (25.03)	
Cytokeratin 19 fragments	Q1	805 (38.70)	388 (14.76)	1,193 (25.34)	<0.001*
	Q2	667 (32.07)	521 (19.82)	1,188 (25.23)	
	Q3	465 (22.36)	703 (26.75)	1,168 (24.81)	
	Q4	143 (6.88)	1016 (38.66)	1,159 (24.62)	
Squamous cell carcinoma-associated antigen	Q1	567 (28.85)	579 (24.07)	1,146 (26.22)	<0.001*
	Q2	501 (25.50)	550 (22.87)	1,051 (24.05)	
	Q3	546 (27.79)	563 (23.41)	1,109 (25.38)	
	Q4	351 (17.86)	713 (29.65)	1,064 (24.35)	
Neuron-specific enolase	Q1	589 (29.64)	570 (22.51)	1,159 (25.65)	<0.001*
	Q2	609 (30.65)	516 (20.38)	1,125 (24.89)	
	Q3	561 (28.23)	558 (22.04)	1,119 (24.76)	
	Q4	228 (11.47)	888 (35.07)	1,116 (24.70)	
CA125	Q1	678 (34.29)	364 (16.64)	1,042 (25.02)	<0.001*
	Q2	646 (32.68)	396 (18.10)	1,042 (25.02)	
	Q3	493 (24.94)	547 (25.00)	1,040 (24.97)	
	Q4	160 (8.09)	881 (40.27)	1,041 (24.99)	

*:P<0.05; Q1, Q2, Q3, Q4 are quartiles shown in eTable 4. PLR: platelet-to-lymphocyte ratio; NLR: neutrophil-to-lymphocyte ratio; A/G: albumin to globulin ratio; ALT/AST: ratio of alanine aminotransferase to aspartate aminotransferase; Lung cancer NOS: the pathological type of lung cancer is not specified; CA125: Carbohydrate antigen 125

© 2023 Wang L et al. *JAMA Network Open.*

eTable 6. Comparison of baseline characteristics of opportunistic screening group and nonopportunistic group before and after the propensity score matching

Characteristic	Before matching			After matching		
	Opportunistic screening group (n = 2251)	Nonopportunistic group (n = 2983)	SMD	Opportunistic screening group (n = 1364)	Nonopportunistic group (n = 1364)	SMD
Age, mean (SD), y	59.9 (10.1)	63.3 (9.4)	0.35	60.5 (10.1)	61.5 (9.5)	0.11
Smoking						
Never	1706 (75.8)	1658 (55.6)		977 (71.6)	971 (71.2)	
Former	322 (14.3)	553 (18.5)	0.48	204 (15.0)	191 (14.0)	0.03
Current	223 (9.9)	772 (25.9)		183 (13.4)	202 (14.8)	
Drinking alcohol	438 (19.5)	1056 (35.4)	0.36	311 (22.8)	321 (23.5)	0.02
Medical insurance						
Urban and rural residents basic medical insurance	864 (38.4)	1760 (59.0)		561 (41.1)	693 (50.8)	
Urban employees basic medical insurance	1254 (55.7)	959 (32.1)	0.25	739 (54.2)	555 (40.7)	0.08
Commercial insurance	112 (5.0)	208 (7.0)		54 (4.0)	93 (6.8)	
Free medical care	21 (0.9)	56 (1.9)		10 (0.7)	23 (1.7)	
Pathology						
Non–small cell lung cancer	2188 (97.2)	2515 (84.3)		1306 (95.7)	1292 (94.7)	
Small cell lung cancer	52 (2.3)	366 (12.3)	0.43	47 (3.4)	68 (5.0)	0.02
Lung cancer NOS	11 (0.5)	102 (3.4)		11 (0.8)	4 (0.3)	
Tumor site						
Right upper lobe	757 (33.6)	861 (28.9)		456 (33.4)	441 (32.3)	
Left upper lobe	567 (25.2)	723 (24.2)		345 (25.3)	332 (24.3)	
Right lower lobe	407 (18.1)	585 (19.6)	0.14	237 (17.4)	256 (18.8)	0.04
Left lower lobe	366 (16.3)	512 (17.2)		230 (16.9)	217 (15.9)	
Right middle lobe	154 (6.8)	302 (10.1)		96 (7.0)	118 (8.7)	
Respiratory diseases	677 (30.1)	1374 (46.1)	0.33	478 (35.0)	489 (35.9)	0.02
Diabetes	315 (14.0)	376 (12.6)	0.04	178 (13.0)	181 (13.3)	0.01
High fluorescence intensity reticulocyte ratio, tertilea						

© 2023 Wang L et al. *JAMA Network Open.*

1	1508 (67.0)	1550 (52.0)		876 (64.2)	820 (60.1)	
2	369 (16.4)	562 (18.8)	0.34	232 (17.0)	268 (19.6)	0.07
3	374 (16.6)	871 (29.2)		256 (18.8)	276 (20.2)	
Eosinophil percentage, quartilea						
1	784 (34.8)	604 (20.2)		428 (31.4)	404 (29.6)	
2	626 (27.8)	629 (21.1)	0.46	368 (27.0)	310 (22.7)	0.09
3	472 (21.0)	840 (28.2)		303 (22.2)	351 (25.7)	
4	369 (16.4)	910 (30.5)		265 (19.4)	299 (21.9)	
Basophil percentage, quartilea						
1	1101 (48.9)	1076 (36.1)		636 (46.6)	630 (46.2)	
2	482 (21.4)	486 (16.3)	0.40	279 (20.5)	241 (17.7)	0.06
3	449 (19.9)	714 (23.9)		280 (20.5)	281 (20.6)	
4	219 (9.7)	707 (23.7)		169 (12.4)	212 (15.5)	
Platelet volume distribution width, quartilea						
1	426 (18.9)	911 (30.5)		273 (20.0)	318 (23.3)	
2	539 (23.9)	787 (26.4)	0.33	342 (25.1)	385 (28.2)	0.11
3	755 (33.5)	833 (27.9)		461 (33.8)	398 (29.2)	
4	531 (23.6)	452 (15.2)		288 (21.1)	263 (19.3)	
Platelet count, quartilea						
1	723 (32.1)	605 (20.3)		421 (30.9)	391 (28.7)	
2	651 (28.9)	650 (21.8)	0.45	376 (27.6)	367 (26.9)	0.08
3	559 (24.8)	752 (25.2)		340 (24.9)	330 (24.2)	
4	318 (14.1)	976 (32.7)		227 (16.6)	276 (20.2)	
Platelet hematocrit, quartilea						
1	759 (33.7)	650 (21.8)		444 (32.6)	383 (28.1)	
2	670 (29.8)	785 (26.3)	0.38	384 (28.2)	414 (30.4)	0.09
3	489 (21.7)	683 (22.9)		303 (22.2)	288 (21.1)	
4	333 (14.8)	865 (29.0)		233 (17.1)	279 (20.5)	
Neutrophil count, quartilea						
1	460 (20.4)	854 (28.6)		311 (22.8)	366 (26.8)	
2	473 (21.0)	838 (28.1)	0.31	303 (22.2)	333 (24.4)	0.12
3	618 (27.5)	687 (23.0)		352 (25.8)	306 (22.4)	
4	700 (31.1)	604 (20.2)		398 (29.2)	359 (26.3)	
NLR, quartilea						

© 2023 Wang L et al. *JAMA Network Open.*

1	451 (20.0)	870 (29.2)	0.35	320 (23.5)	378 (27.7)	0.12
2	471 (20.9)	827 (27.7)		296 (21.7)	300 (22.0)	
3	589 (26.2)	722 (24.2)		322 (23.6)	327 (24.0)	
4	740 (32.9)	564 (18.9)		426 (31.2)	359 (26.3)	
Urea, quartilea						
1	541 (24.0)	852 (28.6)	0.14	329 (24.1)	343 (25.1)	0.05
2	529 (23.5)	768 (25.7)		311 (22.8)	349 (25.6)	
3	563 (25.0)	672 (22.5)		351 (25.7)	315 (23.1)	
4	618 (27.5)	691 (23.2)		373 (27.3)	357 (26.2)	
Albumin and globulin, quartilea						
1	355 (15.8)	977 (32.8)	0.34	249 (18.3)	280 (20.5)	0.04
2	607 (27.0)	729 (24.4)		355 (26.0)	356 (26.1)	
3	662 (29.4)	623 (20.9)		384 (28.2)	341 (25.0)	
4	627 (27.9)	654 (21.9)		376 (27.6)	387 (28.4)	
Alkaline phosphatase, quartilea						
1	837 (37.2)	475 (15.9)	0.63	459 (33.7)	377 (27.6)	0.12
2	644 (28.6)	717 (24.0)		388 (28.4)	404 (29.6)	
3	470 (20.9)	787 (26.4)		298 (21.8)	331 (24.3)	
4	300 (13.3)	1004 (33.7)		219 (16.1)	252 (18.5)	
Globulin, quartilea						
1	727 (32.3)	612 (20.5)	0.45	424 (31.1)	388 (28.4)	0.08
2	658 (29.2)	656 (22.0)		381 (27.9)	378 (27.7)	
3	540 (24.0)	739 (24.8)		327 (24.0)	319 (23.4)	
4	326 (14.5)	976 (32.7)		232 (17.0)	279 (20.5)	
Total bilirubin, quartilea						
1	414 (18.4)	905 (30.3)	0.32	275 (20.2)	296 (21.7)	0.04
2	544 (24.2)	760 (25.5)		338 (24.8)	337 (24.7)	
3	613 (27.2)	708 (23.7)		369 (27.1)	363 (26.6)	
4	680 (30.2)	610 (20.4)		382 (28.0)	368 (27.0)	
Total bile acids, quartilea						
1	806 (35.8)	658 (22.1)	0.36	470 (34.5)	423 (31.0)	0.07
2	518 (23.0)	638 (21.4)		300 (22.0)	316 (23.2)	
3	523 (23.2)	833 (27.9)		333 (24.4)	339 (24.9)	
4	404 (17.9)	854 (28.6)		261 (19.1)	286 (21.0)	
Carcinoembryonic antigen, quartilea						
1	809 (35.9)	508 (17.0)	0.70	421 (30.9)	384 (28.2)	0.13

© 2023 Wang L et al. *JAMA Network Open.*

2	701 (31.1)	602 (20.2)		415 (30.4)	351 (25.7)	
3	493 (21.9)	812 (27.2)		321 (23.5)	376 (27.6)	
4	248 (11.0)	1061 (35.6)		207 (15.2)	253 (18.5)	
Cytokeratin 19 fragments, quartile[a]						
1	928 (41.2)	489 (16.4)	0.87	473 (34.7)	428 (31.4)	0.07
2	672 (29.9)	601 (20.1)		393 (28.8)	412 (30.2)	
3	487 (21.6)	764 (25.6)		351 (25.7)	344 (25.2)	
4	164 (7.3)	1129 (37.8)		147 (10.8)	180 (13.2)	
Neuron-specific enolase, quartile[a]						
1	671 (29.8)	656 (22.0)	0.45	399 (29.3)	397 (29.1)	0.07
2	677 (30.1)	620 (20.8)		393 (28.8)	372 (27.3)	
3	652 (29.0)	672 (22.5)		399 (29.3)	349 (25.6)	
4	251 (11.2)	1035 (34.7)		173 (12.7)	246 (18.0)	
Cancer antigen 125, quartile[a]						
1	803 (35.7)	508 (17.0)	0.81	406 (29.8)	405 (29.7)	0.07
2	742 (33.0)	565 (18.9)		438 (32.1)	407 (29.8)	
3	553 (24.6)	755 (25.3)		383 (28.1)	352 (25.8)	
4	153 (6.8)	1155 (38.7)		137 (10.0)	200 (14.7)	
Chlorine, quartile[a]						
1	479 (21.3)	1032 (34.6)	0.37	326 (23.9)	347 (25.4)	0.07
2	436 (19.4)	673 (22.6)		260 (19.1)	277 (20.3)	
3	718 (31.9)	784 (26.3)		422 (30.9)	434 (31.8)	
4	618 (27.5)	494 (16.6)		356 (26.1)	306 (22.4)	
Prothrombin time, quartile[a]						
1	825 (36.7)	634 (21.3)	0.54	472 (34.6)	415 (30.4)	0.10
2	666 (29.6)	652 (21.9)		382 (28.0)	388 (28.4)	
3	477 (21.2)	703 (23.6)		313 (22.9)	323 (23.7)	
4	283 (12.6)	994 (33.3)		197 (14.4)	238 (17.4)	

SMD, standardized mean difference; NLR, neutrophil-to-lymphocyte ratio.

[a]Quartiles and tertiles are shown in eTable 4.

© 2023 Wang L et al. *JAMA Network Open.*

eTable 7. Variable names from the top line to the bottom line in eFigure 3(A)

**Variables from the top line to the bottom line**	**Coefficients**
Diabetes	0.406
Medical insurance	0.286
Skin conditions	0.211
Total bilirubin	0.209
Sex	0.203
Basophil count	0.138
Large platelet ratio	0.138
Chronic nephritis and kidney disease	0.120
Platelet volume distribution width	0.114
Chlorine	0.102
Total protein	0.087
Aspartate aminotransferase	0.084
Nationality	0.080
Adenosine deaminase	0.065
Urea	0.062
NLR	0.059
Family history of lung cancer	0.055
White blood cell count	0.051
Percentage of reticulocytes	0.047
Albumin	0.047
Average red blood cell volume	0.043
Red blood cell distribution width cv	0.036
Eosinophil count	0.034
Creatinine	0.032
Lymphocyte count	0.031
Urea nitrogen creatinine ratio	0.029
Average amount of red blood cell hemoglobin	0.024
PLR	0.022
A/G	0.022
Red blood cell distribution width sd	0.006
ALT/AST	0.005
Monocytes count	0
Low fluorescence intensity reticulocyte ratio	0
Platelet count	0
Red blood cell count	0
Alanine aminotransferase	0
International normalized ratio	-0.007
Hemoglobin amount	-0.007

© 2023 Wang L et al. *JAMA Network Open.*

Age	-0.007
Sodium	-0.010
Uric acid	-0.013
Glucose	-0.016
Average red blood cell hemoglobin concentration	-0.021
Calcium	-0.023
Potassium	-0.023
Monocytes ratio	-0.024
Percentage of eosinophils	-0.029
Digestive diseases	-0.032
Direct bilirubin	-0.038
Percentage of neutrophils	-0.042
γ-glutamyl transpeptidase	-0.046
Percentage of lymphocytes	-0.051
Neutrophil count	-0.053
Reticulocyte count	-0.057
Total bile acids	-0.061
Squamous cell carcinoma-associated antigen	-0.062
High fluorescence intensity reticulocyte ratio	-0.065
Platelet hematocrit	-0.067
Hematocrit	-0.071
Medium fluorescence intensity reticulocyte ratio	-0.072
Tumor location	-0.074
Prothrombin activity	-0.078
Indirect bilirubin	-0.083
Globulin	-0.098
Cardiovascular diseases	-0.099
Neuron-specific enolase	-0.105
Alkaline phosphatase	-0.134
Average platelet volume	-0.138
Carcinoembryonic antigen	-0.241
Smoking	-0.242
Drinking alcohol	-0.253
Prothrombin time	-0.303
Cytokeratin 19 fragments	-0.312
Basophil percentage	-0.312
Cancer antigen 125	-0.313
Marriage	-0.355
Anemia of chronic disease	-0.394
Respiratory diseases	-0.419
Diffuse connective tissue disease	-0.504

© 2023 Wang L et al. *JAMA Network Open.*

Pathology	-0.665

PLR: platelet-to-lymphocyte ratio; ALT/AST: ratio of alanine aminotransferase to aspartate aminotransferase; NLR: neutrophil-to-lymphocyte ratio; A/G: albumin to globulin ratio; CA125: Carbohydrate antigen 125

© 2023 Wang L et al. *JAMA Network Open.*

eTable 8. Propensity score analysis stratified by TNM stage

TNM stage	Total num with complete screening information (Opportunistic screening)	Variables from PSM	Num after PSM	HR (95%CI), P for lung cancer death<br>HR (95%CI), P for all-cause death
stage I	2499 (1729)	Age,<br>Medical insurance,<br>Respiratory diseases,<br>Platelet volume distribution width,<br>Carcinoembryonic antigen	1434	Before PSM:<br>0.28 (0.16-0.47), p<0.001<br>0.39 (0.25-0.60), p<0.001<br>PSM:<br>0.45 (0.25-0.83), p=0.010<br>0.58 (0.35-0.96), p=0.033<br>PSRA:<br>0.36 (0.21-0.61), p<0.001<br>0.48 (0.31-0.74), p=0.001<br>IPTW:<br>0.36 (0.21-0.63), p<0.001<br>0.50 (0.32-0.78), p=0.003
stage II	293 (132)	Smoking,<br>Respiratory diseases,<br>Globulin,<br>Cancer antigen 125	156	Before PSM:<br>0.43 (0.23-0.79), p=0.007<br>0.45 (0.25-0.79), p=0.006<br>PSM:<br>0.99 (0.47-2.13), p=0.989<br>1.00 (0.48-2.09), p=0.991<br>PSRA:<br>0.67 (0.35-1.26), p=0.211<br>0.68 (0.37-1.23), p=0.201<br>IPTW:<br>0.64 (0.31-1.29), p=0.209<br>0.63 (0.32-1.24), p=0.178
stage III	781 (216)	Smoking,<br>Drinking alcohol,<br>Pathology,<br>Respiratory diseases,<br>Large platelet ratio,<br>Basophil percentage,<br>Direct bilirubin,<br>Total bilirubin,<br>Cytokeratin 19 fragments,<br>Cancer antigen 125,<br>Potassium,	328	Before PSM:<br>0.44 (0.33-0.58), p<0.001<br>0.45 (0.35-0.59), p<0.001<br>PSM:<br>0.65 (0.46-0.93), p=0.017<br>0.67 (0.48-0.94), p=0.021<br>PSRA:<br>0.72 (0.54-0.97), p=0.032<br>0.74 (0.56-0.99), p=0.040<br>IPTW:<br>0.75 (0.53-1.06), p=0.100

© 2023 Wang L et al. *JAMA Network Open.*

		Prothrombin activity,<br>Prothrombin time		0.78 (0.56-1.09), p=0.146
stage IV	1449 (133)	Albumin,<br>Total protein,<br>Cytokeratin 19 fragments,<br>Cancer antigen 125	264	Before PSM:<br>0.62 (0.49-0.79), p<0.001<br>0.63 (0.50-0.79), p<0.001<br>PSM:<br>0.74 (0.54-1.01), p=0.058<br>0.75 (0.55-1.02), p=0.067<br>PSRA:<br>0.75 (0.59-0.96), p=0.024<br>0.76 (0.60-0.97), p=0.024<br>IPTW:<br>0.82 (0.62-1.08), p=0.163<br>0.84 (0.65-1.08), p=0.173

PSM: propensity score matching; PSRA: propensity score regression adjusting; IPTW: inverse propensity treatment weighing

© 2023 Wang L et al. *JAMA Network Open.*

eTable 9. Results of HR for 2-year case fatality and length-bias correction factor correcting for lead time and length biases

Lead time (Days)	HR (95%CI) after correction for lead time bias	Median HR (range) after additional correction of length-bias	Length-bias correction factor
Lung cancer death			
150	0.15 (0.13-0.18)	0.15 (0.15-0.18)	1.001
180	0.16 (0.13-0.19)	0.16 (0.15-0.18)	1.026
210	0.16 (0.14-0.19)	0.16 (0.16-0.19)	0.993
240	0.17 (0.15-0.21)	0.18 (0.18-0.21)	1.038
270	0.18 (0.15-0.21)	0.19 (0.18-0.22)	1.051
All-cause death			
150	0.16 (0.14-0.19)	0.17 (0.16-0.19)	1.046
180	0.17 (0.14-0.20)	0.17 (0.17-0.20)	1.008
210	0.17 (0.15-0.20)	0.18 (0.17-0.20)	1.034
240	0.19 (0.16-0.22)	0.19 (0.19-0.22)	1.020
270	0.19 (0.17-0.23)	0.20 (0.20-0.23)	1.032

Length-bias correction factor: Median HR after correction for length-bias / HR before correction for length-bias

© 2023 Wang L et al. *JAMA Network Open.*

eTable 10. Propensity score analysis after correcting possible lead time and length biases by multiplying length-bias correction factor

Lead time (Days)	HR (95%CI), *P* for lung cancer death<br>HR (95%CI), *P* for all-cause death	Corrected HR (95%CI) for lung cancer death<br>Corrected HR (95%CI) for all-cause death
150	Before PSM:<br>0.16 (0.14-0.19), p<0.001<br>0.18 (0.15-0.21), p<0.001<br>PSM:<br>0.62 (0.51-0.75), p<0.001<br>0.65 (0.54-0.78), p<0.001<br>PSM*:<br>0.68 (0.56-0.82), p<0.001<br>0.70 (0.59-0.84), p<0.001<br>PSRA:<br>0.64 (0.54-0.75), p<0.001<br>0.67 (0.57-0.78), p<0.001<br>IPTW:<br>0.77 (0.63-0.95), p=0.015<br>0.79 (0.65-0.96), p=0.017	Before PSM:<br>0.16 (0.14-0.19)<br>0.19 (0.16-0.21)<br>PSM:<br>0.62 (0.51-0.75)<br>0.68 (0.57-0.82)<br>PSM*:<br>0.68 (0.56-0.82)<br>0.73 (0.61-0.88)<br>PSRA:<br>0.64 (0.54-0.75)<br>0.70 (0.59-0.82)<br>IPTW:<br>0.77 (0.63-0.95)<br>0.83 (0.68-1.00)
180	Before PSM:<br>0.17 (0.14-0.20), p<0.001<br>0.18 (0.16-0.21), p<0.001<br>PSM:<br>0.64 (0.53-0.78), p<0.001<br>0.67 (0.56-0.81), p<0.001<br>PSM*:<br>0.70 (0.58-0.85), p<0.001<br>0.72 (0.60-0.87), p<0.001<br>PSRA:<br>0.66 (0.55-0.78), p<0.001<br>0.69 (0.59-0.81), p<0.001<br>IPTW:<br>0.80 (0.65-0.99), p=0.037<br>0.82 (0.68-0.99), p=0.044	Before PSM:<br>0.17 (0.15-0.20)<br>0.18 (0.16-0.21)<br>PSM:<br>0.66 (0.54-0.80)<br>0.68 (0.57-0.81)<br>PSM*:<br>0.72 (0.59-0.87)<br>0.73 (0.61-0.87)<br>PSRA:<br>0.67 (0.57-0.80)<br>0.69 (0.59-0.81)<br>IPTW:<br>0.82 (0.67-1.01)<br>0.83 (0.68-1.00)
210	Before PSM:<br>0.17 (0.15-0.20), p<0.001<br>0.19 (0.16-0.22), p<0.001<br>PSM:<br>0.66 (0.55-0.80), p<0.001<br>0.69 (0.58-0.83), p<0.001	Before PSM:<br>0.17 (0.15-0.20)<br>0.20 (0.17-0.23)<br>PSM:<br>0.66 (0.54-0.80)<br>0.72 (0.60-0.86)

© 2023 Wang L et al. *JAMA Network Open.*

	PSM*:	PSM*:
	0.72 (0.59-0.87), p<0.001	0.71 (0.59-0.87)
	0.75 (0.62-0.90), p<0.001	0.77 (0.64-0.93)
	PSRA:	PSRA:
	0.68 (0.57-0.80), p<0.001	0.67 (0.57-0.80)
	0.71 (0.60-0.83), p<0.001	0.73 (0.63-0.86)
	IPTW:	IPTW:
	0.83 (0.68-1.02), p=0.081	0.83 (0.67-1.02)
	0.85 (0.70-1.03), p=0.097	0.88 (0.72-1.07)
240	Before PSM:	Before PSM:
	0.18 (0.15-0.21), p<0.001	0.19 (0.16-0.22)
	0.19 (0.17-0.23), p<0.001	0.20 (0.17-0.23)
	PSM:	PSM:
	0.69 (0.57-0.83), p<0.001	0.71 (0.59-0.86)
	0.72 (0.60-0.86), p<0.001	0.73 (0.61-0.88)
	PSM*:	PSM*:
	0.74 (0.61-0.90), p=0.003	0.77 (0.64-0.94)
	0.77 (0.64-0.92), p=0.005	0.79 (0.66-0.94)
	PSRA:	PSRA:
	0.70 (0.59-0.83), p<0.001	0.73 (0.61-0.86)
	0.73 (0.62-0.86), p<0.001	0.75 (0.64-0.87)
	IPTW:	IPTW:
	0.86 (0.70-1.06), p=0.157	0.89 (0.73-1.10)
	0.88 (0.72-1.07), p=0.190	0.90 (0.74-1.09)
270	Before PSM:	Before PSM:
	0.19 (0.16-0.22), p<0.001	0.20 (0.17-0.23)
	0.20 (0.17-0.23), p<0.001	0.21 (0.18-0.24)
	PSM:	PSM:
	0.71 (0.59-0.86), p<0.001	0.75 (0.62-0.90)
	0.74 (0.62-0.89), p=0.001	0.77 (0.64-0.92)
	PSM*:	PSM*:
	0.77 (0.63-0.93), p=0.008	0.81 (0.67-0.98)
	0.80 (0.66-0.96), p=0.015	0.82 (0.69-0.99)
	PSRA:	PSRA:
	0.72 (0.61-0.86), p<0.001	0.76 (0.64-0.90)
	0.76 (0.64-0.89), p<0.001	0.78 (0.67-0.91)
	IPTW:	IPTW:
	0.89 (0.73-1.10), p=0.285	0.94 (0.76-1.15)
	0.91 (0.75-1.10), p=0.340	0.94 (0.77-1.14)

PSM: propensity score matching; PSM*: adjusted variables with SMD > 0.10; PSRA: propensity score regression adjusting; IPTW: inverse propensity treatment weighing;

© 2023 Wang L et al. *JAMA Network Open.*

Corrected HR: HR before correction for length-bias × length-bias correction factor (eTable 9)

© 2023 Wang L et al. *JAMA Network Open.*

SUPPLEMENTARY FIGURES

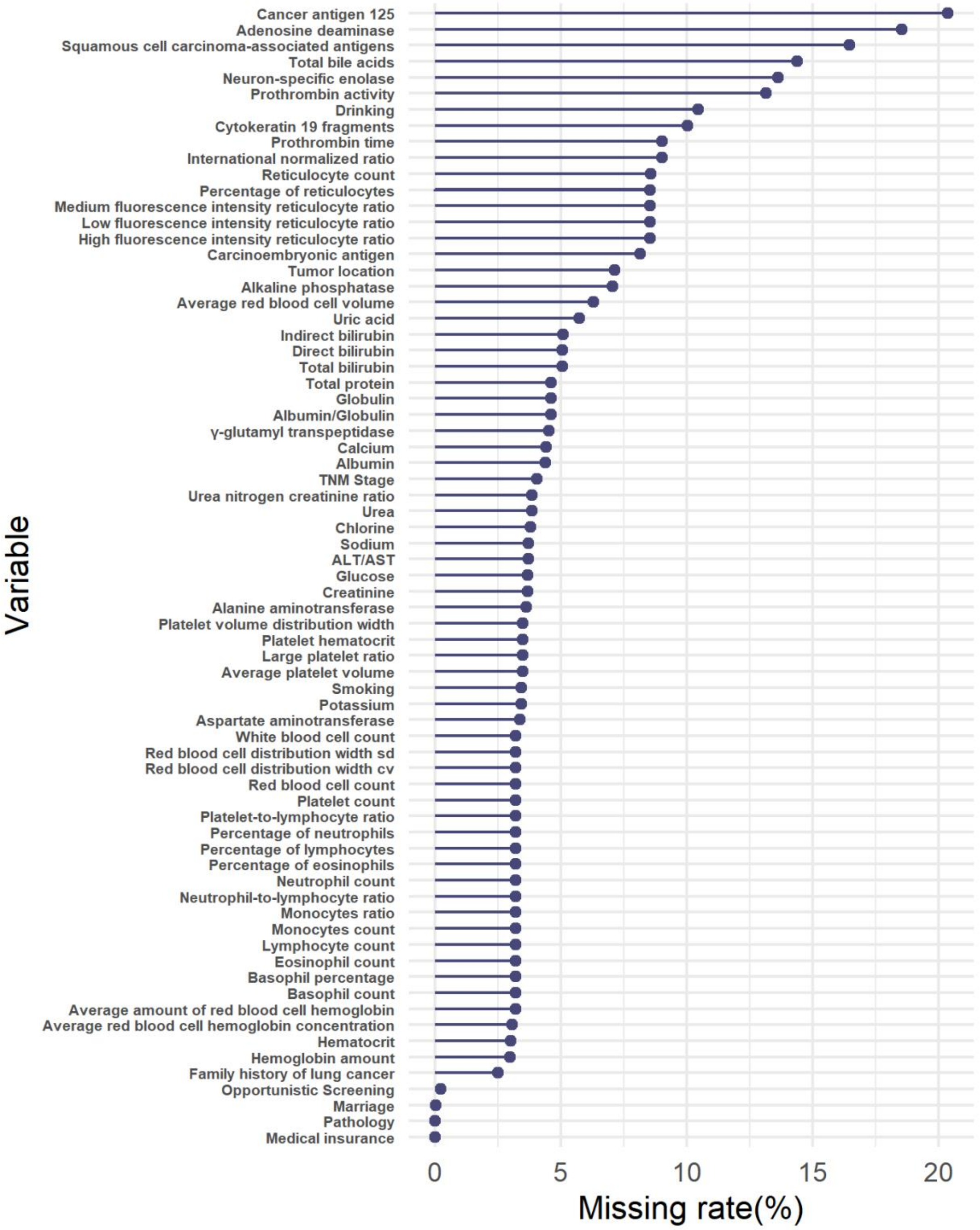

eFigure 1. Missing rate of the variables selected in the study cohort

© 2023 Wang L et al. *JAMA Network Open.*

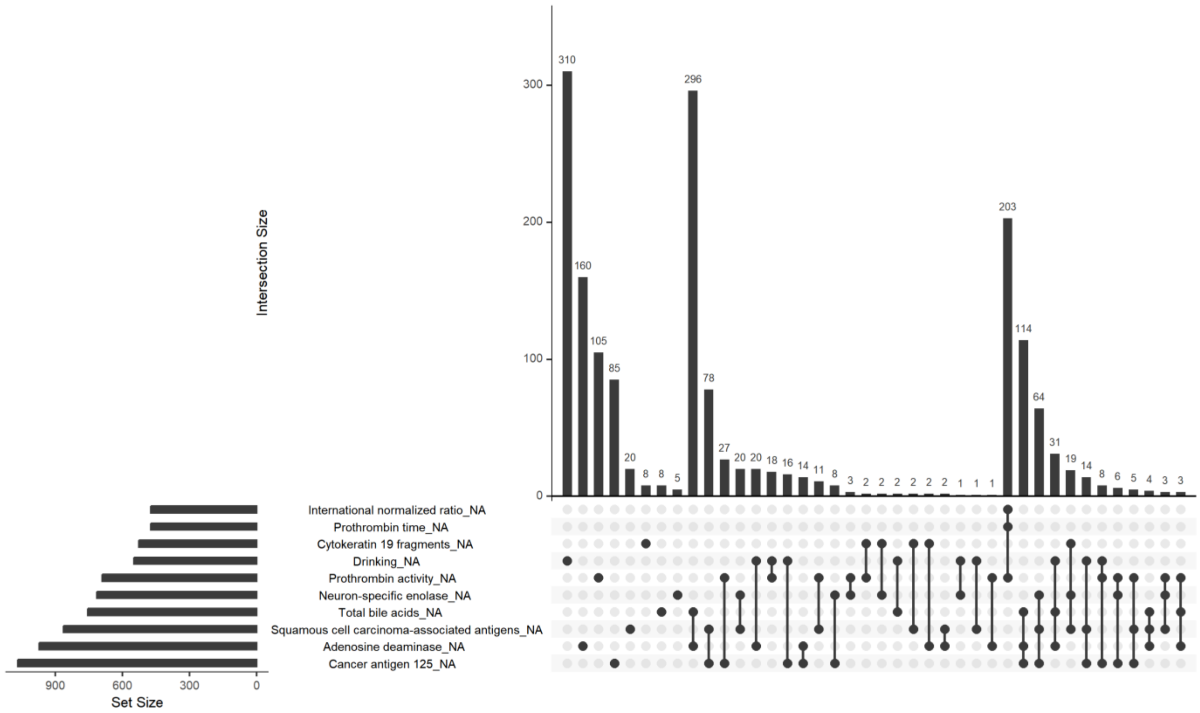

eFigure 2. The patterns of missingness for the top 10 variables with the most missingness

© 2023 Wang L et al. *JAMA Network Open.*

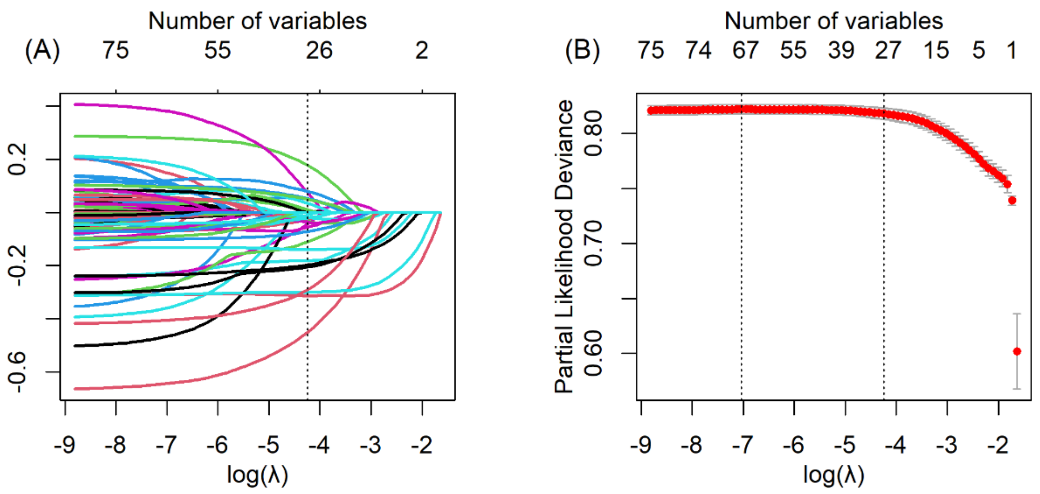

eFigure 3. Variable selection using the LASSO logistic regression model. In eFigure 3 (A), each curve represents the change trajectory of each variable's coefficient distribution. The Y-axis is the value of the coefficients, the lower row is log(λ), and the upper row is the number of non-zero coefficients in the model. Dotted vertical lines represents the optimized hyperparameter λ by 10-fold cross-validation, which ensured that the model had the minimum deviance. The optimal λ is 0.01437711. Variable names from the top line to the bottom line in eFigure 3 (A) is shown in eTable 6. In eFigure 3 (B), partial likelihood deviance was generated against the log (λ) sequence. Left dotted vertical line represents the optimal λ values by using the 1 standard error of the minimum criteria (the 1-SE criteria), while right dotted vertical line was drawn using the minimum criteria that minimizes the partial log-likelihood deviance

© 2023 Wang L et al. *JAMA Network Open.*

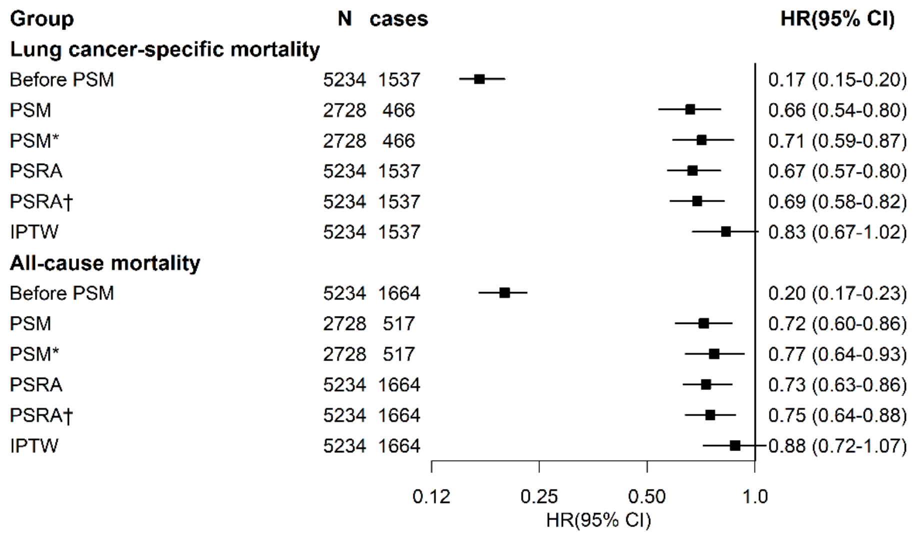

eFigure 4. Forest plot of Cox regression model estimates for the association between opportunistic LDCT screening with lung cancer-specific death and all-cause death using PSM (propensity score matching), PSRA (propensity score regression adjusting) and IPTW (inverse propensity treatment weighing) after correction for lead time (210 days) and length biases. *: PSM adjusted variables with SMD > 0.10, †: PSRA adjusted variables with SMD > 0.10

© 2023 Wang L et al. *JAMA Network Open.*

References

[1] S M, F K, J Y, R C, Yh S, S T, Ja D, Hb E S. Effectiveness of surveillance for hepatocellular carcinoma in clinical practice: A United States cohort[J]. Journal of hepatology, 2016, 65(6).

[2] Qiao E M, Voora R S, Nalawade V, Kotha N V, Qian A S, Nelson T J, Durkin M, Vitzthum L K, Murphy J D, Stewart T F, Rose B S. Evaluating the clinical trends and benefits of low-dose computed tomography in lung cancer patients[J]. Cancer Medicine, 2021, 10(20): 7289-7297.

[3] van Meer S, de Man R A, Coenraad M J, Sprengers D, van Nieuwkerk K M J, Klümpen H J, Jansen P L M, IJzermans J N M, van Oijen M G H, Siersema P D, van Erpecum K J. Surveillance for hepatocellular carcinoma is associated with increased survival: Results from a large cohort in the Netherlands[J]. Journal of Hepatology, 2015, 63(5): 1156-1163.

[4] Liu R, Pérez A, Wu D. Estimation of Lead Time via Low-Dose CT in the National Lung Screening Trial[J]. Journal of Healthcare Informatics Research, 2018, 2(4): 353-366.

[5] Yang R, He M, Wang D, Ye R, Li L, Deng R, Shah M, Yeung S C J. Association of cancer screening and residing in a coal-polluted East Asian region with overall survival of lung cancer patients: a retrospective cohort study[J]. Scientific Reports, 2020, 10(1): 17432.

[6] Duffy S W, Nagtegaal I D, Wallis M, Cafferty F H, Houssami N, Warwick J, Allgood P C, Kearins O, Tappenden N, O'Sullivan E, Lawrence G. Correcting for lead time and length bias in estimating the effect of screen detection on cancer survival[J]. American Journal of Epidemiology, 2008, 168(1): 98-104.

© 2023 Wang L et al. *JAMA Network Open.*
