# Supplementary material for: Opportunistic Screening With Low-Dose Computed Tomography and Lung Cancer Mortality in China
Source: JAMA Netw Open. 2023 Dec 12;6(12):e2347176. doi: 10.1001/jamanetworkopen.2023.47176 (PMC10716726; doi:10.1001/jamanetworkopen.2023.47176)
Supplement: Supplement 4. — Data Sharing Statement [file jamanetwopen-e2347176-s004.pdf]

Data Sharing Statement

Wang. Opportunistic Screening With Low-Dose Computed Tomography and Lung Cancer Mortality in China. *JAMA Netw Open*. Published December 12, 2023.
doi:10.1001/jamanetworkopen.2023.47176

Data
Data available: No
